# Eliminating the need for anodic gas separation in CO_2_ electroreduction systems via liquid-to-liquid anodic upgrading

**DOI:** 10.1038/s41467-022-30677-x

**Published:** 2022-06-02

**Authors:** Ke Xie, Adnan Ozden, Rui Kai Miao, Yuhang Li, David Sinton, Edward H. Sargent

**Affiliations:** 1 Department of Electrical and Computer Engineering, University of Toronto, 10 King’s College Road, Toronto, ON M5S 3G4 Canada; 2 Department of Mechanical and Industrial Engineering, University of Toronto, 5 King’s College Road, Toronto, ON M5S 3G8 Canada

**Keywords:** Carbon capture and storage, Chemical engineering, Energy, Energy efficiency

## Abstract

Electrochemical reduction of CO_2_ to multi-carbon products (C_2+_), when powered using renewable electricity, offers a route to valuable chemicals and fuels. In conventional neutral-media CO_2_-to-C_2+_ devices, as much as 70% of input CO_2_ crosses the cell and mixes with oxygen produced at the anode. Recovering CO_2_ from this stream adds a significant energy penalty. Here we demonstrate that using a liquid-to-liquid anodic process enables the recovery of crossed-over CO_2_ via facile gas-liquid separation without additional energy input: the anode tail gas is directly fed into the cathodic input, along with fresh CO_2_ feedstock. We report a system exhibiting a low full-cell voltage of 1.9 V and total carbon efficiency of 48%, enabling 262 GJ/ton ethylene, a 46% reduction in energy intensity compared to state-of-art single-stage CO_2_-to-C_2+_ devices. The strategy is compatible with today’s highest-efficiency electrolyzers and CO_2_ catalysts that function optimally in neutral and alkaline electrolytes.

## Abstract (web summary)

In the electrified conversion of CO2 to multicarbon products, CO2 crossover to the O2-rich anodic stream adds a further, energy-intensive, chemical separation step. Here, the authors demonstrate a strategy that eliminates the separation requirement.

## Introduction

The electrochemical conversion of CO_2_ (CO_2_RR) to multi-carbon (C_2+_) products is a promising approach to reducing net CO_2_ emissions^1^. The best existing CO_2_RR flow cell systems^2,3^ and zero-gap membrane electrode assembly (MEA) systems^4–6^ achieve C_2+_ Faradaic efficiencies (FEs) of 70% and C_2+_ partial current densities of over 1 A cm^−2^ in flow cells and over 100 mA cm^−2^ in MEAs. These productivity levels are in a regime of interest with respect to industrial application^7^.

Nevertheless, the total energy required for present-day CO_2_-to-C_2+_ electrolysis is too high—for example, when targeting ethylene, today’s electrosynthesis systems require fully 8× more energy to produce ethylene than is embodied in the product^8,9^. Major energy costs are incurred in the electrolyser and the downstream separation steps (see Methods: Energy assessment and Supplementary note 1 in SI)^7,9^. Established approaches to reducing the electrolysis energy requirements include increasing the selectivity for the target product^5,6^ and incorporating alternative anode reactions^1,10–13^. The major energy and economic penalties associated with downstream separation of CO_2_ remain a challenge^14^.

Downstream separation is required to isolate products and recover unconverted CO_2_ from the product streams and electrolytes^9^. Recovering CO_2_ is particularly costly, requiring 25% and 70% of total energy input in the case of neutral and alkaline media CO_2_-to-C_2+_ electrolysers, respectively^8,15^. Present-day CO_2_RR catalysts operate with highly alkaline local conditions (pH > 12) to promote C_2+_ generation at the cathode^2^. However, the carbonate-forming side reaction (CO_2_ + OH^−^ → CO_3_^2−^ or HCO_3_^−^) is favored under alkaline conditions, consuming the majority of the CO_2_ injected^4,6,15^.

**Fig. 1:**
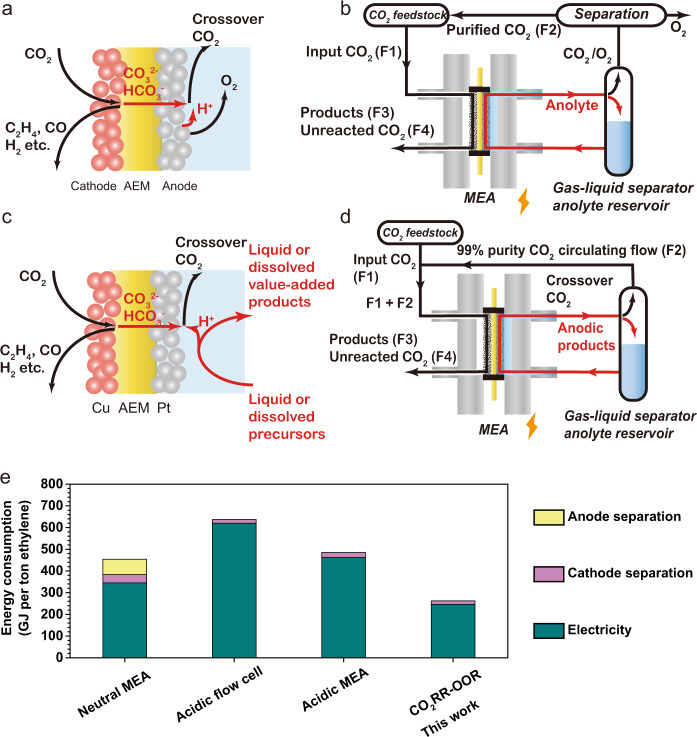
All-liquid anode enabling CO_2_ recycling and low energy intensity ethylene. **a** Mass balance of the electrochemical process in the conventional CO_2_RR-OER electrolysers. **b** Operating principle of the conventional CO_2_RR-OER electrolysis. F1, F2, F3, and F4 are the CO_2_ that: is input, crosses over, is converted to products, and is unreacted, respectively. **c** Mass balance of the electrochemical process in the CO_2_RR-OOR MEA electrolyser. The MEA-type electrolyser uses a Cu-loaded gas diffusion electrode as the cathode, a Pt/C loaded hydrophilic carbon cloth as the anode, and an anion-exchange membrane (AEM) as the solid-state electrolyte. At the cathode, some CO_2_ is electrochemically converted to CO_2_RR products, and a significant fraction of CO_2_ is converted to carbonate/bicarbonate due to the high local pH. The carbonate/bicarbonate ions then migrate to the anode through the AEM. An organic precursor is electrochemically oxidized to value-added product(s) in a near-neutral electrolyte and generates protons at the anode. The protons combine with the carbonate/bicarbonate ions, regenerating CO_2_ as the only gas-phase product at the anodic product stream. **d** The operating principle of the CO_2_RR-OOR electrolysis system combines low-energy input and high-carbon utilization in CO_2_-to-C_2+_ conversion. The system uses an anolyte composed of KHCO_3_ and liquid organic precursors. The cathode chamber is fed with humidified CO_2_. The mass balance table is provided as Table S1. **e** Energy intensity of ethylene production in benchmark systems from literature (Neutral MEA^4^; acidic flow cell^14^; acidic MEA^21^) versus this work.

Operating with neutral electrolytes (e.g., KHCO_3_) in a membrane electrode assembly cell mitigates CO_2_ loss. However, a significant amount of input CO_2_ (~3× more than the fraction that converted to C_2+_) crosses the anion exchange membrane (AEM) to the anode as carbonate/bicarbonate, combining with the protons generated from the anodic reaction and is converted back to CO_2_ (Fig. 1a)^4,15^.

On the anode side, crossover CO_2_ combines with O_2_ produced via the oxygen evolution reaction (OER) to produce a gas mixture of 60–80% CO_2_ and 20–40% O_2_^16,17^. This mixture cannot be directly recycled into the cathode because oxygen reduction would dominate at the cathode^18^. As a result, separation of CO_2_ is required downstream of the anode, incurring an energy penalty of an added 50–100 GJ per ton of ethylene produced (see Supplementary note 1)—an energy cost greater than the energy consumption of petrochemical ethylene production (67.5 GJ ton^−1^)^19^.

Recent energy and techno-economic assessments demonstrated that this level of separation penalty associated with anodic CO_2_ recovery is prohibitive^9,15,20^. Operating under acidic conditions^14,21,22^ offers one emerging strategy to eliminate CO_2_ crossover and the associated anodic separation energy. However, for now at least, this approach incurs high energy costs in the electrolysis step linked to the high full-cell voltage and low C_2+_ FE in an acidic environment (Fig. 1e, Table 1): today the energy intensity of producing ethylene in acidic CO_2_RR electrolysers is higher than that in conventional AEM-based electrolysers (Fig. 1e, Table 1). The further effort needs to be devoted to developing new catalysts and systems for addressing the voltage, selectivity, and stability problems caused by the acidic environment.

**Table 1:** Energy assessment comparison between the state-of-art CO_2_-to-ethylene CO_2_RR devices.

Metrics	Neutral-MEA^a^ ^4^	Acidic flow cell^a^ ^14^	Acidic MEA^a^ ^21^	This work (max carbon efficiency)	This work (max FE)	This work (min energy)
Reaction pair	CO_2_R-OER	CO_2_R-OER	CO_2_R-OER	CO_2_R-GOR	CO_2_R-GOR	CO_2_R-GOR
Cell type	MEA	Flow cell	MEA	MEA	MEA	MEA
Electrolyte	Neutral	Acidic	Acidic	Neutral	Neutral	Neutral
Full-cell voltage (V)	3.75	4.20	3.80	1.90	1.80	1.90
Ethylene FE (%)	45	28	34	26	45	32
Anode O_2_ FE (%)	100	100	100	─	0.6	─
Anode GOR FE (%)	0	0	0	─	94^e^	─
Current density (mA cm^−2^)^f^	120	1200	100	100	100	100
Input CO_2_ flow rate (sccm cm^−2^)	10	3	0.8	0.18	10	0.36
CO_2_ carbon efficiency (%)	3.6	78	31	78	N/A	48
CO_2_-to-ethylene carbon efficiency (%)	1.2	28.4	10.5	36	1.1	23
Demonstrated stability (h)	100^b^	14^c^	8^d^	─	─	80^b^
**Energy distributions (GJ per ton ethylene)**						
Electrolyser electricity	345	620	462	302	165	246
Cathode gas separation	38	17	23	17	147	16
Anode gas separation	71	0	0	0	0	0
**Overall energy**	**499**	**637**	**485**	**319**	**312**	**262**

^a^All the data sets from references are the ones that consume the least overall energy for producing one ton of ethylene.

^b^Recorded with the CO_2_ carbon efficiency indicated in the same column above.

^c^Recorded with a CO_2_ carbon efficiency of 20%.

^d^Recorded with a CO_2_ carbon efficiency of 1.8%.

^e^The sum of the GOR product FEs obtained from NMR (Fig. 3e) and HPLC (Fig. S13).

^f^The comparison between CO_2_RR-OER and CO_2_RR-GOR systems can be found in Table S14 of SI.

We pursued a strategy that enables the direct recovery of CO_2_ from the anodic gas stream without energy penalty. As in prior works, the cathode chamber of the MEA electrolyser is continuously supplied with CO_2_. The anode chamber employs a near-neutral anolyte (e.g., 1 M KHCO_3_) containing a liquid-phase precursor to be electrochemically oxidized. The AEM separates cathode and anode and provides the locally alkaline conditions favorable for CO_2_RR. In this system, some of the input CO_2_ (flow rate: F1 mol s^−1^) crosses over to the anode; some is converted to products (F3); some passes the MEA (F4) with the products. A key feature of the system is the fact that the anode outlet gas can be recirculated (F2) as an inlet gas to the cathode. The carbon efficiency is defined as:^14^

1 $${{{{{\rm{Carbon}}}}}}\,{{{{{\rm{efficiency}}}}}}\,\left( \% \right)=\frac{F3}{F1}\times 100 \%$$

In an ordinary AEM-based, zero-gap CO_2_RR-OER electrolyser (Fig. 1a, b), a large portion of input carbon (F2, roughly triple to F3^4,6^) crosses over from the cathode to the anode and combines with the protons from the anodic process to regenerate gaseous CO_2_. Without recirculation, this system shows a carbon efficiency (<30%) close to that of ordinary MEA electrolysers.

We noted that if the anodic reaction is designed to be all-liquid in nature— i.e., if it avoids O_2_ evolution—then the approach avoids contamination of this CO_2_ stream with O_2_. The recovered CO_2_ is of high purity (>99%), enabling direct recycling to the cathode (Fig. 1d), i.e., *F1* = *F3* + *F4*. Such a system breaks the 25% CO_2_ utilization limit^15^ in AEM-based electrolyzers, avoids the full energy penalty associated with anodic gas separation, and does so without incurring penalties to full-cell voltage or selectivity to ethylene^7^.

The approach requires an all-liquid-phase anodic process that produces protons (or consumes hydroxides) and operates in near-neutral media. Candidate anode reactions include water-to-hydrogen peroxide^23,24^, chloride-to-hypochlorite^25^, and a wide range of organic oxidation reactions (OORs)^13,26,27^. However, previously-published catalysts for hydrogen peroxide and hypochlorite production result in gaseous by-products^23–25^.

Here we adopt the organic oxidation reaction at the anode. Recent techno-economic analyses^1,27^ have suggested substituting OER with OOR as an opportunity to reduce full-cell voltage and produce salable products from both sides of the cell. Candidate OOR reactions include the oxidation of glucose^1^, glycerol^1^, furfural^27^, 5-hydroxymethylfurfural^28^, alcohols^10–12^ or any other biomass polyols and simple sugars. The coupling of CO_2_RR and the oxidation of organics has been demonstrated in H-cells^10–12,28,29^, liquid-fed flow cells^30^, gas-fed flow cells^1,13^, and MEA electrolysers^31^. Coupling electrochemical CO reduction with OOR has been recently demonstrated in an MEA electrolyser^8^. However, prior systems that employed OOR as an anodic process did not focus on overall carbon efficiency: recent gas-CO_2_-fed CO_2_RR-OOR systems operated in strong alkaline electrolytes (pH > 14)^1,13,31^, causing a severe energy penalty associated with the regeneration of (bi)carbonate back to alkaline and CO_2_.

Glucose is abundant from biomass, with an average market price of $400-500 ton^−1^ ^32^, mainly produced from starch. In 2017, over 5 million tons of glucose were produced in the United States (https://www.statista.com/statistics/496485/glucose-production-in-the-us/). Electrochemical oxidation of glucose produces gluconate, glucuronate, and glucarate (Fig. S1)^33,34^, and these are feedstocks for the production of biopolymers^35^ and pharmaceuticals^36^. Gluconic acid commands a market price of $700–1600/ton^32,37,38^. The projected market size of gluconic acid is $80 million^39^ (2024). Recent techno-economic assessments estimated that^32,40^ renewable energy-powered electrochemical glucose oxidation reaction (GOR) is economically feasible. GOR outcompetes OER at industrially relevant reaction rates in neutral and near-neutral electrolytes^41^. GOR also offers electrolysis energy savings, with a thermodynamic potential of 0.05 V^1^, significantly lower than that of OER (1.23 V).

In this work, we couple the CO_2_RR with the glucose oxidation reaction (GOR) and demonstrate the liquid-phase anodic process strategy. The system shows a low full-cell voltage of 1.9 V and total carbon efficiency of 48%, enabling 262 GJ/ton ethylene, a 46% reduction in energy intensity compared to state-of-art single-stage CO_2_-to-C_2+_ devices.

## Results

### Catalyst characterization

**Fig. 2:**
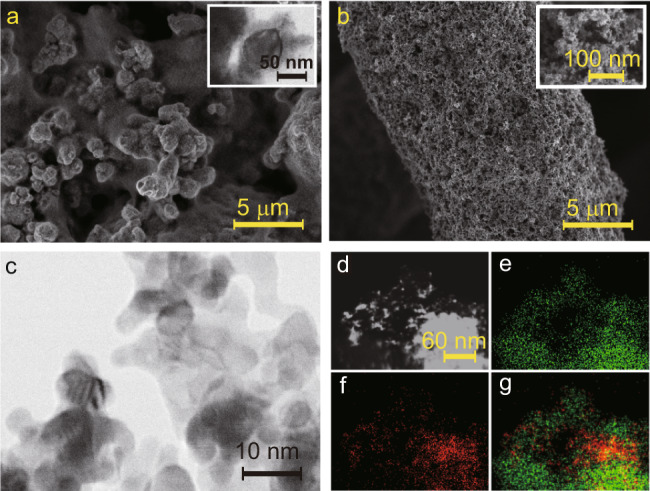
Electron microscopy characterization of MEA electrolyser catalysts. **a** Scanning electron microscopy (SEM) and transmission electron microscopy (TEM, inset) images of the cathodic catalyst: Cu nanoparticles/PFSA composite. **b** SEM images of the anodic catalyst: Pt/C loaded on hydrophilic carbon fibers. **c** SEM images of the Pt/C catalyst. **d**–**g** The scanning transmission electron microscopy image (**d**) and corresponding Energy-dispersive X-ray spectroscopy (EDS) elemental mappings of carbon (**e**), platinum (**f**), and overlap (**g**) for Pt/C catalyst.

To perform CO_2_RR on the cathode, we deposited Cu nanoparticles and perfluorosulfonic acid (PFSA) ionomer on a hydrophobic porous polytetrafluoroethylene (PTFE) gas diffusion electrode. The PTFE gas diffusion electrode was pre-sputtered with 200 nm-thick polycrystalline Cu to improve electrical conductivity (see Methods for details). Scanning and transmission electron microscopy (SEM and TEM, respectively) images reveal a surface morphology composed of Cu nanoparticles bonded by several tens of nm-thick PFSA ionomer films (Fig. 2a). The anode electrode comprised a homogeneous blend of Pt/C nanoparticles and PFSA ionomer on a hydrophilic and highly porous carbon fiber cloth substrate (see Methods for further details). SEM images confirm that the anode is composed of macroporous carbon fibers (Fig. 2b) that are homogeneously covered by Pt/C nanoparticles and PFSA composites (inset in Fig. 2b). TEM images in Fig. 2c show that the diameter of Pt nanoparticles is in the range 5–10 nm. Energy-dispersive X-ray spectroscopy (EDS) elemental mapping reveals that Pt is evenly distributed on the surface of C nanoparticles (Fig. 2e and g).

### Electrochemical characterization of the CO_2_RR-GOR system

**Fig. 3:**
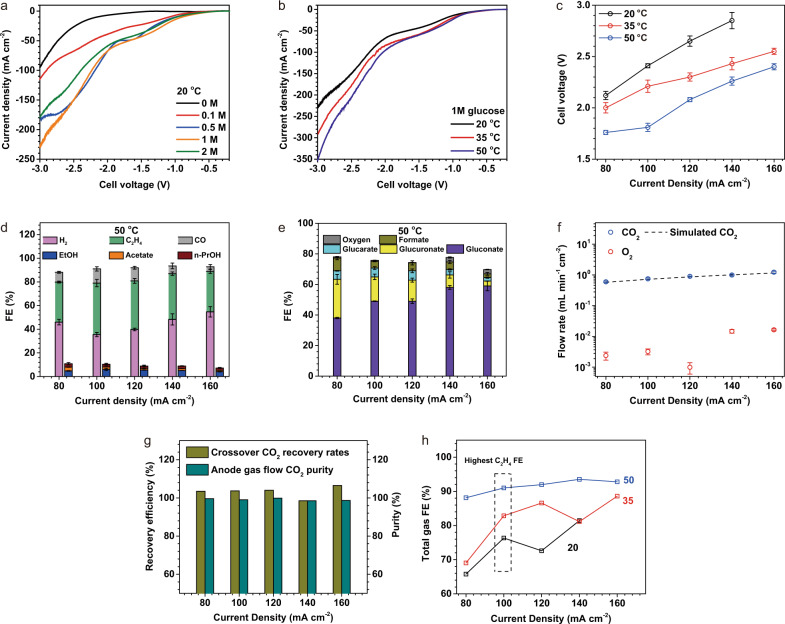
Performance of the CO_2_RR-GOR electrolysis system. The mass loadings on the cathode and anode are Cu: 0.5 mg cm^−2^ and Pt: 2 mg cm^−2^. **a**, **b** The linear scan voltammetry (LSV) of the CO_2_RR-GOR electrolysis system with various glucose concentrations (0 M refers to CO_2_RR-OER on a high-surface-area IrO_x_-Ti catalyst) at 20 ^o^C (**a**) and (**b**) with 1 M glucose at various temperatures. All the profiles were recorded at a scanning rate of 5 mV s^−1^ immediately after three cycles of voltammetry scanning. **c** The full-cell potentials of the CO_2_RR-GOR at various temperatures which are measured by applying constant currents. **d** CO_2_RR FE distributions at 50 ^o^C and a number of different current densities. The liquid FEs are based on the sum of products detected from cathodic + anolyte outlets. **e** FE distributions of liquid products (measured by NMR and GC) of GOR at various current densities at 50 ^o^C. GOR products were also quantified using HPLC, see Fig. S13. **f** The CO_2_ and O_2_ flow rates (normalized by electrode geometric area) in the anodic gas streams at 50 ^o^C. The simulated CO_2_ is assessed by the stoichiometry of generated OH^-^ and transferred electrons, assuming CO_2_ is converted to CO_3_^2-^. **g** Recovery efficiency and purity of CO_2_ at the anodic product stream at various current densities and 50 ^o^C. Recovery efficiency is defined by dividing the CO_2_ flow rate from measurement by that from prediction. **h** The FE distributions of gas products of CO_2_RR at various temperatures and current densities.

We conducted linear scan voltammetry (LSV) measurements to investigate the electrochemical response of the CO_2_RR-GOR system with a cathode Cu loading of 0.5 mg cm^−2^ and an anode Pt loading of 2 mg cm^−2^ (Fig. 3a and b). When we did not add glucose to the anolyte (CO_2_RR-OER), the electrolyser delivered a current density of 94 mA cm^−2^ at a full-cell voltage of 3 V. At 100 mA cm^−2^, when we introduced glucose, increasing its concentration in the anolyte from 0.1 to 0.5 M and 1 M, the full-cell voltage decreased from 2.90 to 2.18 V and 2.23 V. The full-cell voltages at glucose concentrations 0.5 M and 1 M are rather close to one another: this we attribute to the electrokinetic limitations of the anode^42^: at 0.5 M, glucose molecules saturate the active sites of the Pt catalyst, and, as a result, increasing the concentration to 1 M does not enable further reduction in the cell voltage. A further increase in the glucose concentration to 2 M increased full-cell voltage, i.e., 2.40 V at 100 mA cm^−2^ due to the excess coverage of Pt with glucose and oxidation intermediates^43^. We thus adopted 1 M glucose for the performance investigations.

Performing LSV and chronopotentiometry measurements, we investigated the voltage-current density dependence at various temperatures (Fig. 3b and c). Elevating the operating temperature from 20 to 35 ^o^C lowers the full-cell voltage by 0.1–0.3 V in a wide range of current densities from 80 to 160 mA cm^−2^ (Fig. 3b and c), attributed to accelerated electrochemical kinetics. A similar full-cell voltage reduction was observed as the operating temperature increased from 35 to 50 ^o^C.

### Achieving high ethylene FE and low oxygen FE simultaneously

Maintaining a low OER FE is critical to ensure high GOR efficiency and sufficient purity of the recovered CO_2_. In the present CO_2_RR-GOR system, we found that the cathodic and anodic catalysts needed to be carefully engineered to enable the CO_2_ recovery strategy.

In prior studies, typical mass loadings of the cathode Cu (CO_2_RR) and anode Pt (GOR) were 1 mg cm^−2^^6,44^ and 0.5 mg cm^−2^^8^. When we used these configurations in our CO_2_RR-GOR system, we obtained high full-cell voltages of >3.4 V when seeking to operate above 100 mA cm^−2^, Fig. S5a, showing little advantage over CO_2_RR-OER systems (Fig. S3). The high full-cell voltage degraded the selectivity of GOR over OER, leading to an anodic O_2_ FE of >8% (Fig. S5b).

Reexamining Pt loading was essential to reduce the full-cell voltage to <2.4 V (Fig. S5a), and consequently the O_2_ FE to <1% (Fig. S5b) at the current density of 120 mA cm^−2^. However, operating at this current density, the CO_2_RR selectivity toward ethylene is ~30% (Fig. S5c, d), significantly lower than the 40–45% benchmark for Cu^4,6^. To achieve this benchmark, the system must run at 200 mA cm^−2^ (Fig. S5d) with the full-cell voltage of 3.23 V and O_2_ FE of 7% (Fig. S5a, b). This is not overcome by further increasing Pt loading (Fig. S5a), as discussed in Fig. S5. Tuning the Cu loading changes the current density required to maximize the ethylene FE. We, therefore, pair a 0.5 mg cm^−2^ Cu cathode with a 2 mg cm^−2^ Pt anode to achieve maximum ethylene FE and low oxygen FE simultaneously at industrial-relevant current densities.

When run at 100 mA cm^−2^ and 50 ^o^C, the CO_2_RR-GOR system provides a full-cell voltage of 1.8 ± 0.1 V, representing a 1.6 V lower voltage than the conventional CO_2_RR-OER system at the same current density and temperature^4^. We attribute this low full-cell voltage to the lower thermodynamic potential of GOR than OER (0.05 vs. 1.23 V) and the anodic catalyst’s high activity toward GOR. Such a low full-cell potential significantly reduces electricity demand (Table 1). At 100 mA cm^−2^, the system delivers ethylene FEs of 42%, 48%, and 44% at 2  ^o^C, 35 ^o^C, and 50 ^o^C (Figs. S7, 3d, Tables S5, S6 and S8). The Cu-sputtered on the PTFE GDE is unlikely to participate in CO_2_RR: we observed similar performance when spraying Cu nanoparticles onto a hydrophobic carbon paper (Fig. S4).

We investigated the selectivity of the GOR in a wide range of current densities (from 80 to 160 mA cm^−2^) and operating temperatures (Fig. 3e, Tables S7 and S9). With the temperature increasing from 20 to 50 ^o^C, we detected a slight increase in anolyte pH (from pH 7.9 to 8.3, Fig. S10), attributable to the lower solubility of CO_2_ in warmer anolyte. We detected gluconate as the major GOR product (>49% FE), achieving a plateau of 58% at 140 mA cm^−2^. The full analysis of GOR products is shown in Figs. S12 and S13. The FEs toward oxygen remained <3% at current densities from 80 and 160 mA cm^−2^ (<1% at 100 mA cm^−2^ and a full-cell voltage of 1.80 V) owing to the sluggish kinetics of OER (Fig. 3a).

### Recycling CO_2_

The CO_2_ recycling strategy requires a high CO_2_ recovery efficiency, in this work defined as:

2 $${{{{{\rm{Recovery}}}}}}\; {{{{{\rm{efficiency}}}}}}=\frac{{{{{{\rm{Detected}}}}}}\,{{{{{\rm{anodic}}}}}}\,{{{{{{\rm{CO}}}}}}}_{2}\,{{{{{\rm{flow}}}}}}\,({{{{{\rm{mol}}}}}}\,{{{{{\rm{s}}}}}}^{-1})}{{{{{{\rm{Theore}}}}}{tical}}\,{{{{{{\rm{CO}}}}}}}_{2}\,{{{{{\rm{crossover}}}}}}\,({{{{{\rm{mol}}}}}}\,{{{{{\rm{s}}}}}}^{-1})}\times 100 \%$$

3 $${{{{{\rm{Theoretical}}}}}}\,{{{{{{\rm{CO}}}}}}}_{2}\,{{{{{\rm{crossover}}}}}}\,({{{{{\rm{mol}}}}}}\,{{{{{\rm{s}}}}}}^{-1})=\frac{I\,(A)}{F(C{{{{{{\rm{mol}}}}}}}^{-1})}\times \frac{1}{2}$$

where *I* refers to the current and *F* the Faraday constant. Here the CO_2_ crossing over to the anode is in the form of carbonate^17^. The experiment suggested that the amount of CO_2_ collected at the anode is in good agreement with the stoichiometry of OH^-^ generated and electrons transferred^17^ (Fig. 3f), indicating a CO_2_ recovery efficiency approaching 100% (Fig. 3g). In addition, the anodic CO_2_ flow rate is three orders of magnitude larger than that of O_2_ (Fig. 3f), indicating a purity of CO_2_ recovered from the anodic gas stream exceeding 99% (Fig. 3g). This low level of O_2_ enables direct recycling of this anode gas stream in the cathode without the need for separation and associated energy costs. Notably, an oxygen fraction of over 1.8% deteriorates the CO_2_RR selectivity, as seen in control studies in Fig. S3). Specifically, the cathodic gas product FE distribution (Fig. 3d) of the CO_2_RR-ORR, with the anodic CO_2_ stream directly fed into the cathode, approaches to within 5% absolute the CO_2_RR-OER electrolyser (Fig. S3). The control experiments (Figs. S13, S14) and ^13^C abundance analysis (Table S4) show that >99.8% of the anodic CO_2_ is not from the overoxidation of glucose. These experimental observations are in good agreement with the mass balance analysis provided in Fig. 1c.

### Avoiding the cathode-side CO_2_RR liquid products crossover

Ethylene production via CO_2_RR is accompanied by liquid-phase products such as ethanol, acetate, and propanol, much of which crosses the membrane to the anode stream^4^. Cathode-to-anode crossover of liquid products is a challenge in CO_2_RR as these products risk oxidation and dilution in the anolyte. With OOR on the anode side, there is the additional risk that cathode-produced liquids will contaminate the liquid anode product stream and the gaseous CO_2_ stream^1,27^. When we increased the temperature from 20 to 50 ^o^C, we found that the FEs toward the major gas products of CO_2_RR (C_2_H_4_ and CO) increased from 48 to 56% at a constant current density of 100 mA cm^−2^ (Figs. S4, 3d and h). This observation agrees with previous studies, attributable to the positive entropy change of the CO_2_RR^45,46^. The FE toward the liquid products of CO_2_RR decreased from 24 to 9%—a trend consistent with a previous report^4^. The higher temperature significantly reduced the crossover of ethanol and n-propanol (Fig. S8) to the anode side, a finding we assign to the increased rate of evaporation into the cathode gas stream. As a result, the weight ratio of the liquid CO_2_RR products to the GOR target products in the anolyte stream was <1% at 50 ^o^C (Fig. S9), in contrast to 1.4% at 35 ^o^C (Fig. S9). In light of the evidence that: (i) the total CO_2_RR FE is close to 100% (Fig. 3d, negligible loss due to anode oxidation) and (ii) the ethanol oxidation signal is absent in the CV profiles of the anolyte (Fig. S14), we conclude the oxidation of the CO_2_RR liquid products at the anode is insignificant. Thus, operating at modestly elevated temperatures benefits the CO_2_RR-GOR system by reducing full-cell voltage and suppressing the formation and crossover of liquid CO_2_RR products.

### CO_2_RR-GOR system performance

Encouraged by these findings, we explored the carbon efficiency upper limits in the CO_2_RR-GOR system. A widely employed approach^14,21^ to determine carbon efficiency upper limits is restricting the CO_2_ availability at the cathodic stream and measuring the ratio between F3 (CO_2_ converted to products) and F1 (CO_2_ feeding). The current and FE distributions determine F2 and F3. F1 is regulated to tune the CO_2_ availability. In the CO_2_RR-GOR system, F1 = F3 + F4, meaning that F4 needs to be suppressed by lowering F1 to achieve high carbon efficiency. In principle, in a given electrolysis system, the relative values of F1, F2, F3, and F4 are proportional to the electrolyser area. We thus normalize all the flow rates by the electrolyser area in this study to focus in on the intrinsic properties of this system.

**Fig. 4:**
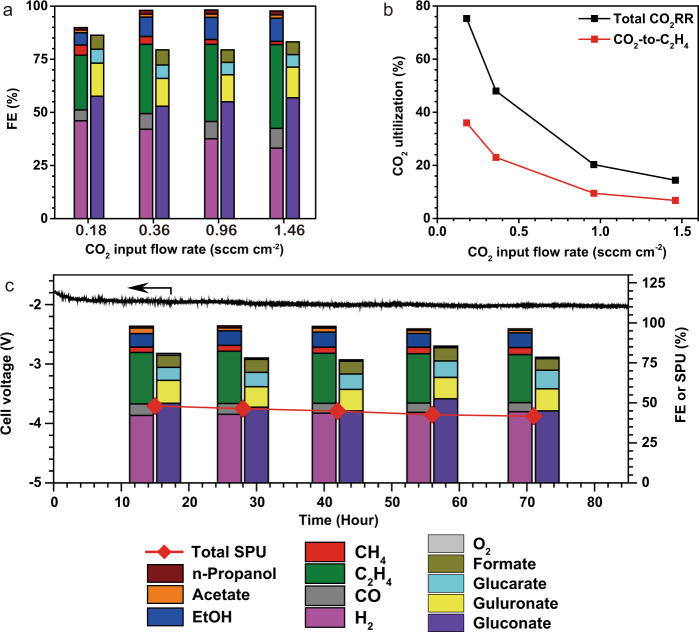
Performance of the CO_2_RR-GOR system under low CO_2_ availability at 50 ^o^C. The mass loadings on the cathode and anode are Cu: 0.5 mg cm^−2^ and Pt: 2 mg cm^−2^. **a** The FE distributions at various CO_2_ input flow rates. The labels are indicated below (**c**). **b** The CO_2_ carbon efficiency for total CO_2_RR and CO_2_-to-C_2_H_4_ (mole ratio of the input CO_2_ converted to C_2_H_4_) at various CO_2_ input flow rates. **c** Long-term electrolysis performance with a CO_2_ input flow rate of 0.36 sccm cm^−2^. The experiments are performed at a current density of 100 mA cm^−2^. The flow rate of the CO_2_ supplied is normalized by the geometric area of the electrodes.

Decreasing the input CO_2_ flow rate increases the carbon efficiency, as is typical in these systems (Table S10 and Fig. 4b). At an inlet CO_2_ flow rate of 0.18 sccm cm^−2^ (flow rates are normalized by electrode area), the system delivered a total C_2+_ FE of ~34% at a constant current density of 100 mA cm^−2^ and a full-cell voltage of 1.90 V, corresponding to a carbon efficiency of 75% toward all CO_2_RR products (total carbon efficiency, Fig. 4a and b), exceeding the upper limit of carbon efficiency in neutral media CO_2_RR electrolysers^4,6,14^. At these conditions the ethylene FE stabilizes at ~26%, corresponding to a CO_2_-to-ethylene carbon efficiency of ~36% (Fig. 4b). This carbon efficiency is 1.4-fold greater than the theoretical upper limit of 25% in CO_2_-to-ethylene conversion in conventional, neutral-media, AEM-based electrolysers^14^. Restricting the flow rate results in a significant increase in the hydrogen FE (Fig. 4a), which we and others^4,6,14^ attribute to the mass transfer limitation of CO_2_. GOR maintains consistent selectivity and productivity at the anode, independent from CO_2_ availability in the cathodic gas stream (Fig. 4a and Table S11).

A trade-off between carbon efficiency and ethylene FE is typical of CO_2_-to-ethylene electrolysis^4,6,14^ (Fig. 4a and b). A higher carbon efficiency reduces the energy demand for cathode separation, but the accompanying decrease in ethylene FE increases the specific electrolyser energy demand. To reconcile these metrics, we assess the total input energy (electricity, cathode separation, and anode separation per ton of ethylene produced) of various CO_2_RR approaches (see “Methods”)^6^. We find that the CO_2_RR-GOR system achieves the lowest energy consumption of 262 GJ per ton ethylene, and achieves this with the input CO_2_ flow rate of 0.36 sccm cm^−2^, a total carbon efficiency of 48% toward all CO_2_RR products. Under this condition, the FE toward C_2+_ and ethylene are 45% and 32%, respectively (Table 1).

Compared to state-of-art conventional CO_2_-to-ethylene systems (i.e., MEAs based on AEM and neutral electrolyte), the CO_2_RR-GOR system eliminates the anodic separation energy (>53 GJ per ton ethylene, Supplementary note 1). The overall energy intensity of ethylene production is ~46% less than the most energy-efficient prior CO_2_RR systems among neutral and acidic CO_2_-to-ethylene electrolysers (Table 1).

### Stability with high carbon efficiency

Stability is a prerequisite for the industrial application of CO_2_RR. However, long-term operation of CO_2_RR with a high carbon efficiency (e.g., CO_2_ carbon efficiency >40%) has not been achieved to date. The best CO_2_ carbon efficiency achieved for a run duration of 100 h was <4%^6^.

We performed extended CO_2_RR under the operating conditions that enable the lowest energy intensity of ethylene production. The CO_2_RR-GOR system achieves stable electrosynthesis of cathodic C_2+_ and anodic products for over 80 h at a current density of 100 mA cm^−2^, comparable to the stability of conventional MEAs^4–6^. The system maintains an average full-cell voltage of 1.90 ± 0.1 V, an average total C_2+_ FE of 42%, and an average carbon efficiency of ~45% toward all CO_2_RR products (Fig. 4c, Table S12). Similarly, we detected stable GOR productivity throughout (Fig. 4c, Table S13). Notably, this CO_2_-to-C_2+_ electrolyser demonstrates high stability while maintaining high carbon efficiency.

## Discussion

We demonstrate that pairing CO_2_RR with an all-liquid anodic reaction in neutral media combines high carbon efficiency and low energy input in the electrosynthesis of renewable chemicals and fuels. One key to implementing this strategy is pairing the catalyst mass loadings on the cathode and anode to maximize CO_2_RR product selectivity and minimize anodic OER selectivity simultaneously. The CO_2_ that crosses to the anode was recovered from the anodic downstream with a high purity of >99%. By returning CO_2_ to the cathodic upstream, this strategy achieves a high CO_2_ conversion of up to 75%. The combined system achieves a low full-cell potential of 1.90 V at a current density of 100 mA cm^−2^ and stable electrosynthesis of C_2+_ products for over 80 h while maintaining a high CO_2_ conversion of 45%. Accounting for the total electricity and downstream separation energy costs, this method achieves a total energy intensity of 262 GJ per ton of ethylene produced, ~46% lower than that of previous CO_2_RR electrolysers. This work contributes a route to high carbon efficiency in CO_2_RR electrolysis.

## Methods

### Materials

Potassium bicarbonate (KHCO_3_, 99.7%), D-glucose (99.5%), copper nanoparticles (25 nm), Nafion^TM^ 1100 W (5 wt.% in a mixture of lower aliphatic alcohols and water) and Pt/C (40 wt.% Pt on Vulcan XC72) were purchased from Sigma Aldrich and used as received. Aquivion D79-25BS ionomer was purchased from Fuel Cell Store. Piperion (40 μm) was used as the anion-exchange membrane, purchased from W7Energy and stored in 0.5 M KOH. The water used in this study was 18 MΩ Milli-Q deionized- (DI-) water.

### Electrodes

For the CO_2_RR, we prepared the gas diffusion electrodes (GDEs) by spray-depositing a catalyst ink dispersing 1 mg mL^−1^ of Cu nanoparticles and 0.25 mg mL^−1^ of Nafion^TM^ 1100 W in methanol onto a PTFE substrate that pre-sputtered with a 200 nm thick polycrystalline Cu layer. The substrate was prepared by sputtering Cu target, at a rate of 1 Å s^−1^ onto a piece of PTFE membrane in a Magnetron sputtering system^5^. The mass loading of Cu nanoparticles on the GDE was tuned between 0.5 and 1.0 mg/cm^2^. The GDEs were dried in the air overnight prior to experiments.

For the GOR anode electrodes, a commercially available Pt/C was first physically mixed with ionomer (Aquivion D79-25BS) in a glass beaker and then sonicated for 1 h. The resulting catalyst ink was then spray coated on both sides of the hydrophilic carbon cloth until the Pt loading of 0.5–3.2 mg cm^−2^ was achieved.

### Characterizations

Scanning electron microscopy (SEM) images of cathode and anode were captured by an FEI Quanta FEG 250 environmental SEM. Transition electron microscopy (TEM) images and elemental mappings were acquired by an FEI Titan 80–300 kV TEM microscope. X-ray photoelectron spectra (XPS) of the electrodes were determined by a model 5600, PerkinElmer using a monochromatic aluminum X-ray source.

### Assembling of the CO_2_RR-GOR system

The MEA set (5 cm^2^) was purchased from Dioxide Materials. A cathode was cut into a 2.5 cm × 2.5 cm piece and placed onto the MEA cathode plate with a flow window with a dimension of 2.23 cm × 2.23 cm. The four edges of the cathode were sealed by copper tapes and then Kapton tapes, and make sure the tapes did not cover the flow window. A Piperion AEM (40 μm, 3 cm × 3 cm) was carefully placed onto the cathode. A gasket with a 2.23 cm × 2.23 cm window was placed on the cathode. The Pt/C loaded carbon cloth anode (2 cm × 2 cm) was placed onto the AEM.

### Electrochemical measurements

All the data in this work were collected with 0.5 mg/cm^2^ Cu (cathode) and 2 mg/cm^2^ Pt (anode) unless otherwise specified. All the performance metrics were recorded after at least 1000 s of stabilization at a specific condition. The full-cell voltages reported in this work are not iR corrected. All the error bars are presented as standard deviation based on three measurements. The potential cut-off was set to 10 V, and the current applied on the cell was in the range of 0.2–1.0 A.

To evaluate the performance of the CO_2_RR-GOR system under different conditions, the cathode side of the MEA was fed with CO_2_ flow (0.18–10  sccm cm^−2^ of electrode area, 10 sccm cm^−2^ if not specified) that comes from both CO_2_ feedstock and anodic gas stream unless specified. The anode side was circulated with a solution containing 1 M KHCO_3_ and glucose with various concentrations (0.1–2 M) at 10 mL/min by a peristaltic pump. A gas-tight glass bottle with four in/out channels (gas inlet, gas outlet, liquid inlet and liquid outlet) was used as the anolyte reservoir and gas-liquid separator. In typical CO_2_RR-GOR performance evaluations, the gas inlet channel was sealed, and the gas outlet channel is connected to a ‘Y’ shape tubing connector.

Since the anolyte reservoir/gas-liquid separator is gas-tight, the CO_2_ pressure between the feedstock stream and anodic stream will eventually be balanced and promote a steady flow rate from both sides. The electrochemical measurements were performed with a potentiostat (Autolab PGSTAT204 with 10 A booster).

The auxiliary system for heating the CO_2_RR-OOR electrolyser is shown schematically in Fig. S15.

To evaluate the performance of the CO_2_RR-OER system, the same setup as the CO_2_RR-GOR system was used, except that the anodic gas stream was vented, and the anolyte had no glucose.

To evaluate the performance of the HER-GOR system, the cathode was replaced by a Pt/C catalyst spray-coated onto a hydrophobic carbon paper with a loading of 1.0 mg cm^−2^. The cathode was fed with 8 sccm cm^−2^ N_2_ to carry out H_2_.

### Product analysis

The CO_2_RR gas products, oxygen, and CO_2_ were analyzed by injecting the gas samples into a gas chromatograph (Perkin Elmer Clarus 590) coupled with a thermal conductivity detector (TCD) and a flame ionization detector (FID). The gas chromatograph was equipped with a Molecular Sieve 5 A Capillary Column and a packed Carboxen-1000 Column with argon as the carrier gas. The volumetric gas flow rates in and out of the cell were measured with a bubble column. The FE of a gas product is calculated as follows:

$${{FE}}_{i}={x}_{i}\times \frac{{VP}}{{RT}}\times \frac{{n}_{i}F}{J}$$

Where *x*_*i*_ is the volume fraction of the gas product *i*, *V* is the MEA cathode outlet gas flow rate in L s^−1^ (measured by a bubble flow meter), *P* is atmosphere pressure 101.325 kPa, *R* is the ideal gas constant 8.314 J mol^−1^ K^−1^, *T* is the room temperature in K, *n*_*i*_ is the number of electrons required to produce one molecule of product *F* is the Faraday Constant 96485 C mol^−1^, and *J* is the total current in A. To analyse the anodic gas stream component, the gas outlet channel of the anolyte reservoir was disconnected from the tubing for circulating to the cathode. A 20 sccm argon flow was input from the ‘gas inlet’ channel of the anolyte reservoir as the carrier gas to promote the accurate analysis of CO_2_ and O_2_ components in the anode gas.

The liquid products from the cathode side of the SC-MEA were collected using a cold trap containing 5 mL 0 ^o^C water. The collected liquid from the cathode side and the anolyte were quantified separately by the proton nuclear magnetic resonance spectroscopy (^1^H NMR) on an Agilent DD2 500 spectrometer in D_2_O using water suppression mode and dimethyl sulfoxide (DMSO) as the internal standard. Typical ^1^H NMR spectra of GOR can be found in Fig. S11. Fresh anolyte was used for each liquid product quantification plot, and the collection duration was 30~60 minutes. The FE of a liquid product is calculated as follows:

$${{FE}}_{i}={m}_{i}\times \frac{{n}_{i}F}{{Jt}}$$

where *m*_*i*_ is the quantity of the liquid product *i* in mole, *t* is the duration of product collection (1800~3600 seconds).

To evaluate the stability of the CO_2_RR-GOR system, the CO_2_RR gas and cathodic product FE were evaluated using the above-mentioned methods. 50 μL of anolyte (the total volume is 10 litres) was periodically (every 15 ~ 20 h) sampled for ^1^H NMR analysis. The quantity of the CO_2_RR liquid products that cross over to anolyte and the GOR liquid products in a certain period were deduced from the concentration increment from the beginning to the end of this period.

The by-products of the GOR were also measured by high-performance liquid chromatography (UltiMate 3000 HPLC) equipped with an Aminex HPX-87H column (Bio-Rad) and a reflective index detector. The eluent is 0.05 M H_2_SO_4_, and the column was kept at 60 ^o^C.

The anodic CO_2_, CO_2_ from the cylinder, KHCO_3_ and glucose were analyzed for δ^13^C at the Geobiology Isotope Laboratory at the University of Toronto using a Finnigan Gas Bench coupled with a Thermo-Finnigan MAT 253 gas source isotope ratio monitoring mass spectrometer^47^. The Finnigan Gas Bench sample tray was heated to 72 °C and loaded with vials of CO_2_ gas samples. Sample vials were first flushed with helium gas. CO_2_ samples from the anodic gas stream and cylinder were directly injected into the vials. The KHCO_3_ sample reacted with phosphoric acid to release CO_2_. The glucose sample (60~300 μg) reacted with 1 mL oxidant solution (100 mL H_2_O + 4.0 g K_2_S_2_O_8_ + 200 mL of 85% H_3_PO_4_) to release CO_2_^48^. Measured carbon isotopes were compared to the reference materials, CaCO_3_ MERCK (in-house standard), IAEA-CO-8, NBS-19 and IAEA-CO-1 (International Atomic Energy Agency Reference Products for Environment and Trade). Carbon isotope data are reported in the standard delta (δ) notation relative to Vienna Pee Dee Belemnite (VPDB).

### Energy assessment

We evaluated the energy consumptions for electrolyser electricity, cathodic separation, and anodic separation in the context of ethylene—the world’s most-produced feedstock. We consider the state-of-the-art CO_2_RR systems from the literature, including neutral MEA electrolysers, acidic flow-cell, and MEA. This consideration is based on the performance metrics, including selectivity, productivity, and full-cell voltage—a combination of them reflects as energy intensity of producing multi-carbon products (i.e., ethylene). The proximity of these performance metrics will help refine the effect of anodic and cathodic separation on the energy requirement for producing ethylene. We summarize the input parameters to the model for all the systems. The majority of these input parameters listed in Table 1 are from literature^9^ and this work. Cathodic gas separation was modeled into two steps: (i) pressure swing adsorption to remove CO_2_; (ii) cryogenic distillation to separate ethylene from hydrogen and CO. We employed one of the most widely used models^20^ (i.e., biogas upgrading) for evaluating the energy cost associated with cathode gas separation. The energy assessment on anodic CO_2_/O_2_ separation is evaluated in Supplementary note 1 of SI.

For acidic flow-cell and MEA electrolysers, we assume no energy cost associated with the anodic separation considering no CO_2_ availability at the anodic gas stream^14^. The separation energy of the anodic GOR products is not included in this assessment. A previous techno-economic assessment^40^ on the electrochemical GOR suggested that the overall process of the electrochemical GOR is economically feasible. We, therefore, assume that the energy consumed for anodic GOR product purification can be fully covered by selling the value-add GOR products and does not include it in the comparison between different CO_2_RR devices.

## Supplementary information

Supplementary Information

## Data Availability

The data generated in this study are provided in Supplementary Information and Source Data file. Source data are provided with this paper.

## Source data

Source Data
