# Supplementary material for: Eliminating the need for anodic gas separation in CO2 electroreduction systems via liquid-to-liquid anodic upgrading
Source: Nat Commun. 2022 Jun 2;13:3070. doi: 10.1038/s41467-022-30677-x (PMC9163163; doi:10.1038/s41467-022-30677-x)
Supplement: Supplementary file 1 — Supplementary Information [file 41467_2022_30677_MOESM1_ESM.pdf]

Eliminating the need for anodic gas separation in CO_2 electroreduction systems via liquid-to-liquid anodic upgrading

Ke Xie [1,3], Adnan Ozden [2,3], Rui Kai Miao [2], Yuhang Li [1], David Sinton, [*2] Edward H. Sargent [*1]

[1]Department of Electrical and Computer Engineering, University of Toronto, 10 King's College Road, Toronto, ON M5S 3G4, Canada

[2]Department of Mechanical and Industrial Engineering, University of Toronto, 5 King's College Road, Toronto, ON M5S 3G8, Canada

[3]These authors contributed equally

[*]Correspondence:

ted.sargent@utoronto.ca (EHS.)

sinton@mie.utoronto.ca (DS.)

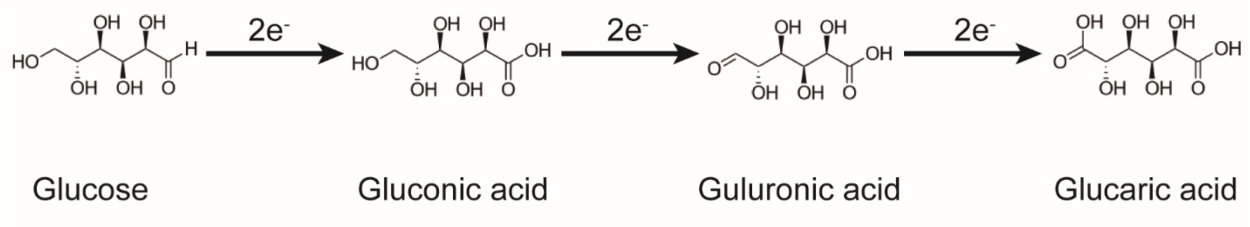

Fig. S1. The major mechanism of the electrochemical glucose oxidation reaction (GOR).

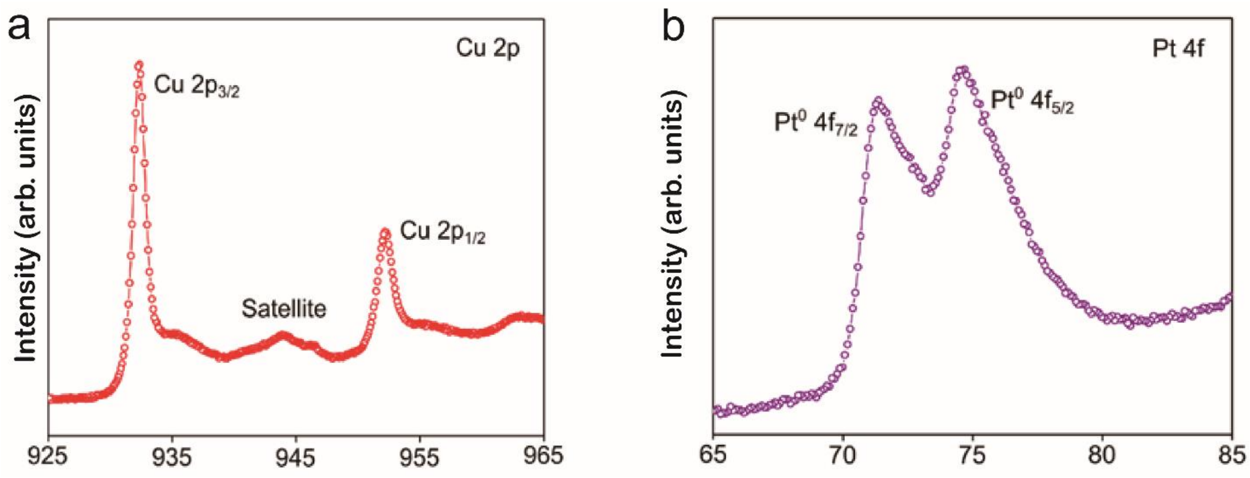

Fig. S2 X-ray photoelectron spectroscopy (XPS) measurements for Cu NPs on Cu/PTFE gas diffusion electrode (a) and Pt-C on hydrophilic carbon paper (b).

Table S1. The CO_2 mass balance of the CO_2RR-GOR system at 50 ^oC at equilibrium. F1, F2, F3 and F4 are the CO_2 of input, crossover, converted and unreacted (Fig. 1d). All are normalized to the equivalent volume flow rates of the CO_2 gas. F2 was measured by GC with F1 of 10 sccm cm^{-2} (Fig. 3f in the main text), assuming it is unchanged with different input CO_2 flow rates. F3 was determined by analyzing the CO_2RR products. F4 was measured by GC. Notably, F2 can be higher than F1 at equilibrium because F2 is the 'dead' CO_2 flow cycling from cathode to anode and back to the cathode.

I (mA cm^{-2})	*F1(sccm cm^{-2})*	*F2(sccm cm^{-2})*	*F3(sccm cm^{-2})*	*F4(sccm cm^{-2})*
100	1.46	0.72	0.21	1.25
100	0.96	0.72	0.19	0.77
100	0.36	0.72	0.17	0.19
100	0.18	0.72	0.14	0.04

Supplementary note 1: the evaluations on the anodic CO_2/O_2 separation energy cost.

Calculation of the separation cost of consuming 1 ton CO_2 in the conventional MEA system due to crossover.

Considering a CO_2RR-OER system would consume 1 ton CO_2 following the process below:

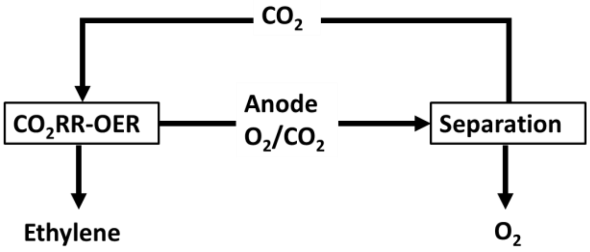

Ideally, CO_2RR only produces ethylene, and no CO_2 passes through the cathode gas chamber. Of 1 ton CO_2 fed into the electrolyser, C_{xo} ton crosses over, separated from O_2 and circulated. 1-C_{xo} ton is converted to ethylene. Repeating this process *n* times until 100% CO_2 is converted to ethylene. The total energy cost (E_{sep}) for separation is:

$$E_{sep} = E_{CO2}\sum_{n=1}^{\infty}(C_{xo})^{\mathrm{n}} = \frac{E_{CO2}}{1-C_{xo}}$$

where E_{CO2} is the energy cost for separating one ton of CO_2 from O_2. In the ideal case, C_{xo} = 0.75: converting 1 ton of CO_2 needs 4 x E_{CO2}.

However, C_{xo} in conventional MEA systems is usually in the range of 0.8~0.9 due to the non-ideal CO_2RR selectivity (30-65% ethylene FE) [1,2]. Converting 1 ton of CO_2 to ethylene needs to separate 5-10 tons of CO_2 from O_2 in total.

The minimum work for CO_2 separation is typically 0.07-0.12 GJ/ton [3]. Assuming 100% second-law efficiency, the separation cost for converting 1 ton CO_2 to ethylene is 0.35-1.2 GJ (1.1-3.7 GJ/ton ethylene). Assuming CO_2 capture plants can achieve a second-law efficiency of ~30% alike the air separation units, the separation costs 1.2-4 GJ, *i.e.* 3.5-13 GJ/ton ethylene.

The real-world CO_2 capture facilities have significantly lower second-law efficiency owing to many factors[3,4] such as heat loss, media regeneration, energy for powering compressor and other auxiliary systems. We summarize the energy cost for capturing CO_2 reported in references in Table S2.

Table S2. The typical energy consumption of various CO_2 capture processes.

Item	Proccess	CO_2 mol fraction (%)	Energy consumption (GJ/ton CO_2)	Reference
1	Monoethanolamine capture	15	3.9	5
2	Monoethanolamine-based HPDSFP process	15	2.3	5
3	Monoethanolamine-based tripled staged flash regeneration	13	4.9	6
4	Cryogenic packed bed*	10	4.0	7
5	Periodic cooled cryogenic packed bed*	15	3.6	8
6	Stirling Cooler system	13	3.4	9
7	Aqueous KOH sorbent coupled to a calcium caustic recovery loop	400 ppm (air)	5.25	10
8	NaOH scrubbing, causticization with lime, thermal calcination in a proposed oxy-blown kiln	400 ppm (air)	7.5	3

* These processes use liquefied natural gas to provide cryogenic energy. The energy cost for liquifying natural gas (LNG) to -162 ^{o}C (0.83 GJ/ton natural gas, 2.6 ton LNG per ton CO_2) is added on top of the operating energy cost.

Depending on the CO_2 mole fraction, source of the input stream and the processes, capturing 1 ton of CO_2 in real-world plants requires 2.3~7.5 GJ. We select the best proximity of CO_2 capture energy based on the following considerations.

The typical capture processes in Table S2 include three types: monoethanolamine-based (1-3 in Table S2), cryogenic-based (4-6 in Table S2) and inorganic alkali-based (7 and 8 in Table S2). Membrane and solid adsorbents-based approaches are less mature[11]. Monoethanolamine suffers from the oxidative degradation induced by O_2, and the CO_2 mole fraction in the target gas input of the alkali-based technique is far below that in the anodic gas stream (~66%). Therefore, we adopt the CO_2 separation energy of 3.4 GJ/ton CO_2 (the lowest) in cryogenic-based approaches for evaluating the anodic CO_2 separation costs.

The separation costs 17-34 GJ for converting 1 ton CO_2 to ethylene, i.e., 53-106 GJ/ton ethylene, in the existing CO_2 capture facilities.

Anodic gas stream CO_2 capture economic assessment

Table S3. The CO_2 capture costs for various processes.

Process	Operation and maintenance ($/ton CO_2)	Capital cost ($/ton CO_2)	Total cost ($/ton CO_2)	Reference
Monoethanolamine absorption	28	16	44	12
Cryogenic	20	30	50	8,11

Inorganic alkali absorption	26	92	118	[10]

Table S3 lists the CO_2 capture costs evaluated for the processes as mentioned above. In principle, producing 1 mol of ethylene causes at least 6 mol CO_2 evolution on the anode, representing 9.4 ton CO_2 per ton ethylene. Considering the nonunity ethylene selectivity (30~65%), the anode evolves 15~30 tons of CO_2 per ton ethylene. Using a cryogenic approach for capturing the anodic CO_2 costs $750~$1500 per ton ethylene. This is prohibitive because the market price of ethylene is $800~$1000 [13].

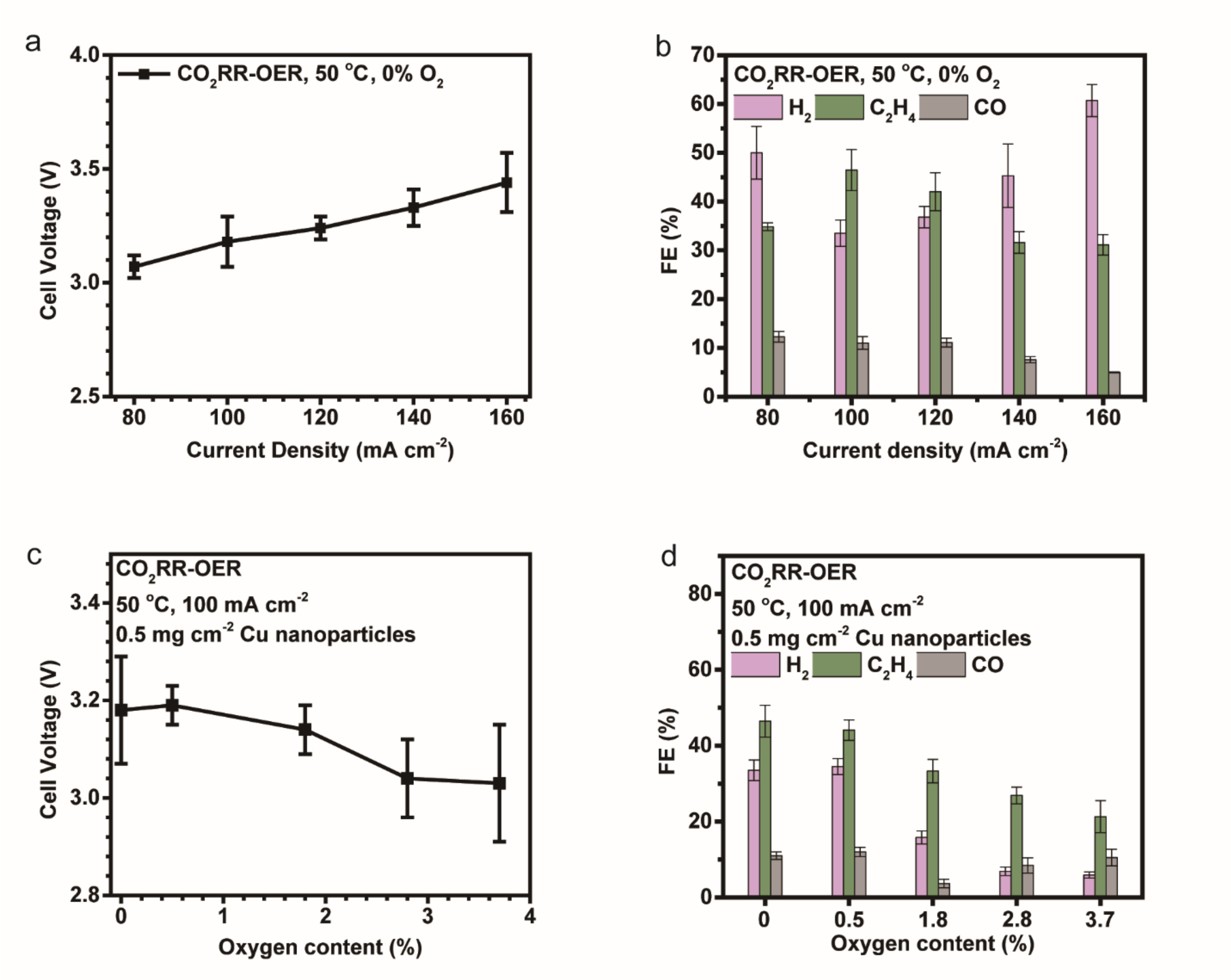

Fig. S3 CO_2RR-OER system performance measured at 50 °C. In all the measurements, the CO_2 input flow rate was kept at 20 sccm cm^{-2}. (a, b) The cell voltages and gas product FEs at different current densities. (c, d) The cell voltages and gas product FEs with different O_2 content in the input CO_2 stream. The O_2 contents were modulated by co-feeding O_2 and CO_2 with certain flow rates and were confirmed by GC. The mass loadings on the cathode and anode are Cu: 0.5 mg cm^{-2} and Pt: 2 mg cm^{-2}.

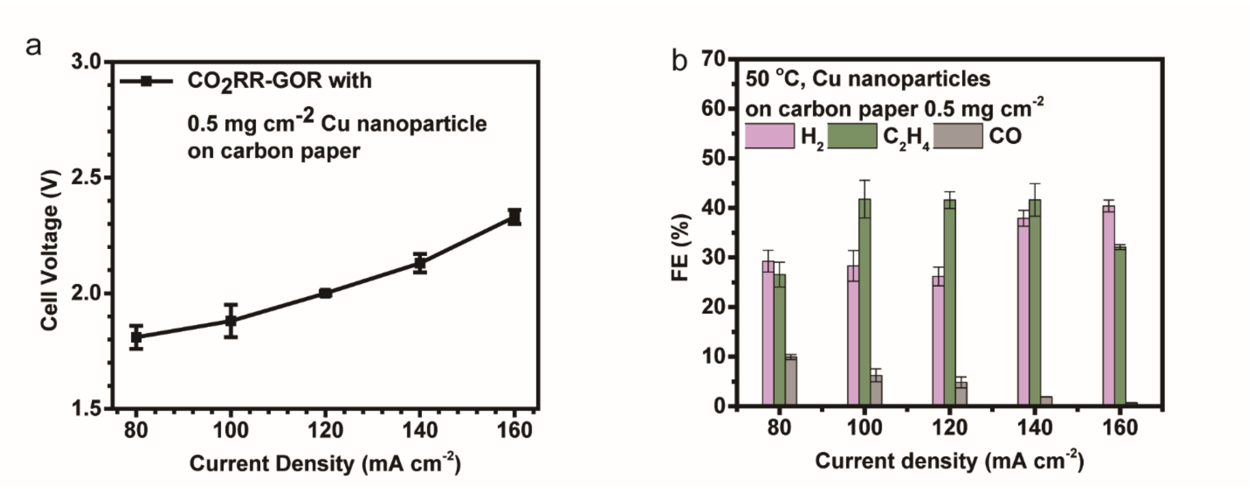

Fig. S4 CO_2RR-GOR system performance measured at 50 °C using Cu/carbon paper catalyst. In all the measurements, the CO_2 input flow rate was kept 10 sccm cm^{-2}. (a, b) The cell voltages and gas product FEs at different current densities. The mass loadings on the cathode and anode are Cu: 0.5 mg cm^{-2} and Pt: 2 mg cm^{-2}.

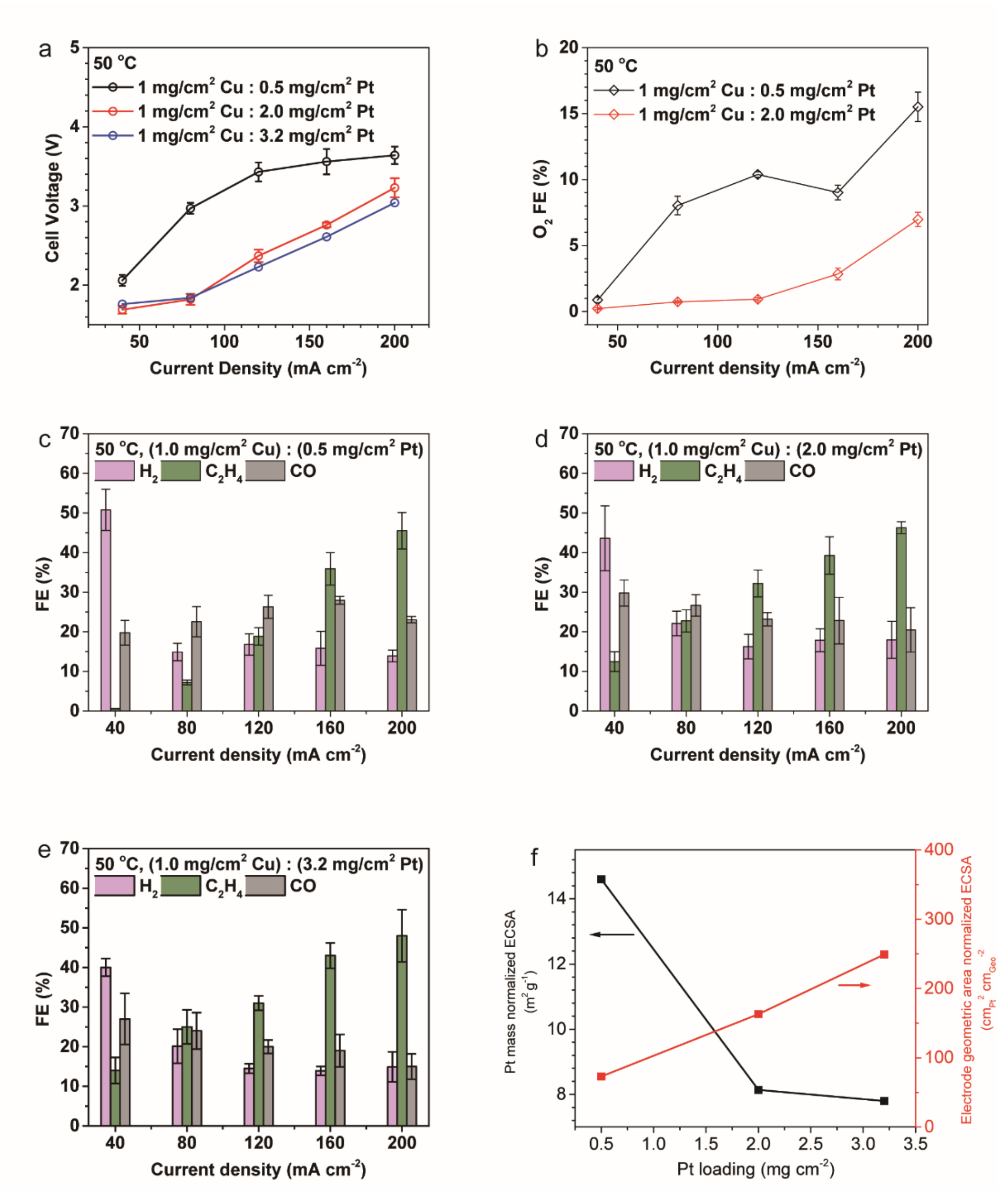

Fig. S5. The performance of the CO_2RR-GOR systems with various cathode and anode catalyst loadings was measured at 50 °C. (a) The dependences of cell voltage on current density. (b) The dependences of oxygen FE on current density. (c-e) The CO_2RR gas product distributions at different current densities for different cathode and anode catalyst loadings as indicated. (f) The ECSAs of the Pt/C anode with various Pt loadings, measured following the protocol suggested in the previous studies[14]. The single-component, three-electrode setup was used, with Pt/C on carbon paper, graphite and Ag/AgCl as the working, counter and reference, respectively. The cyclic voltammetry was recorded at a sweep rate of 5 mV s^{-1} between ~1.0 to ~0 V vs. RHE in Ar-saturated and protected 1 M $KHCO_3$. The hydrogen adsorption/desorption region between ~0 V and ~0.4 V vs. RHE was used for calculation, assuming a charge density of 210 μC/cm^2 Pt.

2 mg cm^{-2} of Pt loading on the anode is close to the typical conventional IrO_2 OER anode used in CO_2RR devices [15,16]. Considering that the market price of Ir is two-fold to Pt [17], such a Pt loading would not induce additional capital costs compared to the conventional CO_2RR devices. In future, non-precious-metal GOR catalysts[18] can be adopted in this system, reducing the associated expenses.

Increasing the anode Pt loading from 0.5 to 2.0 mg cm^{-2} lowers the cell voltage by up to 1.2 V (Fig. S5a), attributing to the increase of anodic active sites (reflected by the electrode geometric area normalized ECSA, Fig. S5f) thus improved electrochemical kinetics. However, further increase the loading to 3.2 mg cm^{-2} has insignificant effect to the cell voltage (Fig. S5a), despite its electrode geometric area normalized ECSA is ~50% higher than 2.0 mg cm^{-2} (Fig. S5f). As such, for the presented CO_2RR-GOR system, we conclude that 2.0 mg cm^{-2} is sufficiently high. The cell voltage is limited by other factors such as the glucose molecule mass transfer efficiency, ohmic resistance, etc.

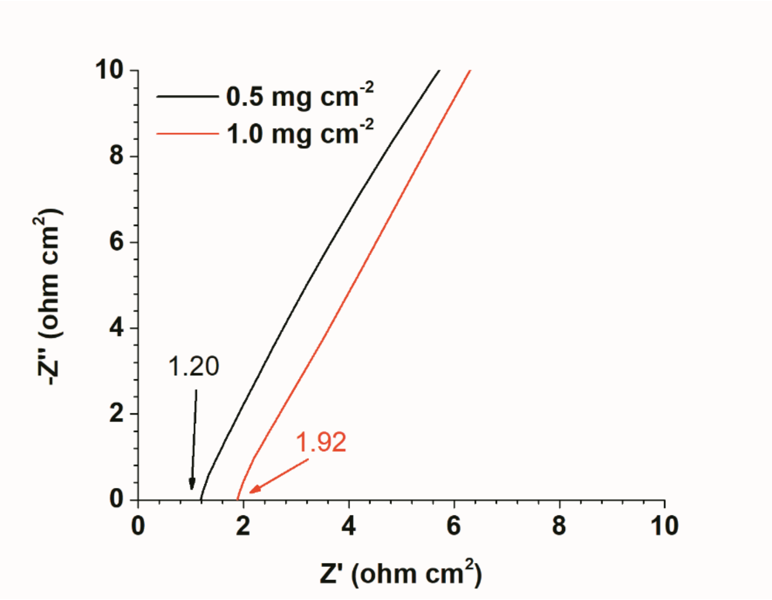

Fig. S6 The Nyquist plots of the <1 mg cm^{-2} Cu : 2 (3.2) mg cm^{-2} Pt> (red) and <0.5 mg cm^{-2} Cu : 2 mg cm^{-2} Pt> (black) MEAs. Z' and Z'' are normailzed by electrode area. The electrochemical impedance spectroscopy (EIS) was performed at 50 ^{o}C, open circuit voltage with a 10 mV amplitude.

The cell voltages of the electrolyzer using <1 mg cm^{-2} Cu : 2 (3.2) mg cm^{-2} Pt> are close to <0.5 mg cm^{-2} Cu : 2 mg cm^{-2} Pt> at the current density of 80 and 100 mA/cm^2, but are higher at the current densities over 120 mA cm^{-2}. The higher ohmic resistance of the former (1.92 vs. 1.20 ohm cm^2, Fig. S6) is responsible to its higher cell voltage.

The electronic conductivity of the cathode relies on the continuous Cu-coated PTFE networks that transfer electrons onto spray-coated Cu nanoparticles. The Cu nanoparticles we spray-coated on the GDE forms a highly porous (non-continuous) layer, with PFSA ionomer coated on the particle surface (see Fig. 2a in the main text). Therefore, the spray-coated Cu nanoparticle layer is less conductive than the Cu-coated PTFE network, and a thicker Cu nanoparticle layer increases the ohmic resistance.

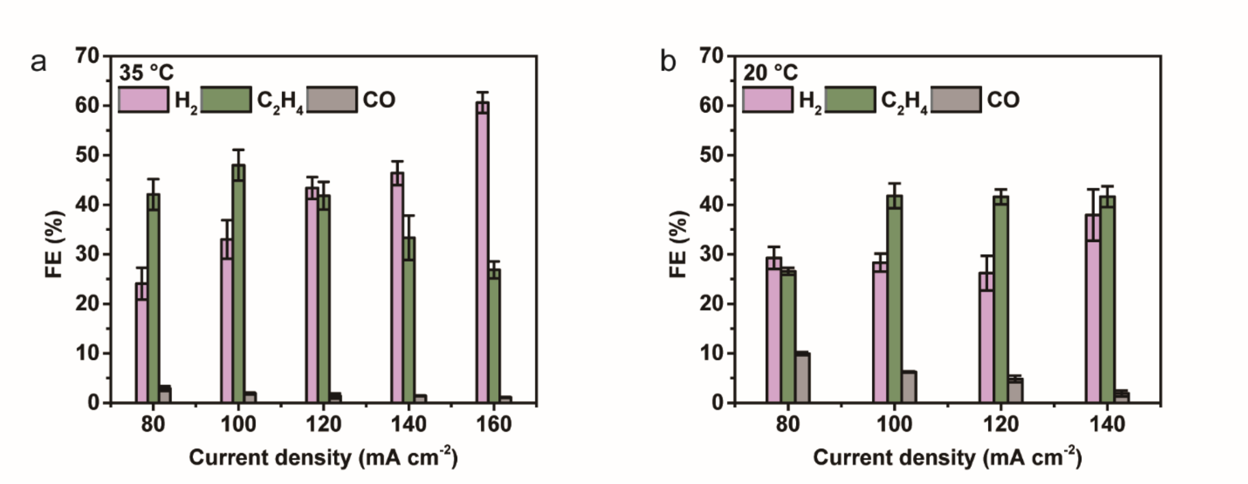

Fig. S7. The FE distributions toward gas-phase CO_2RR products at various current densities. (a) Measured at 35 °C. (b) Measured at 20 °C. The mass loadings on the cathode and anode are Cu: 0.5 mg cm^{-2} and Pt: 2 mg cm^{-2}.

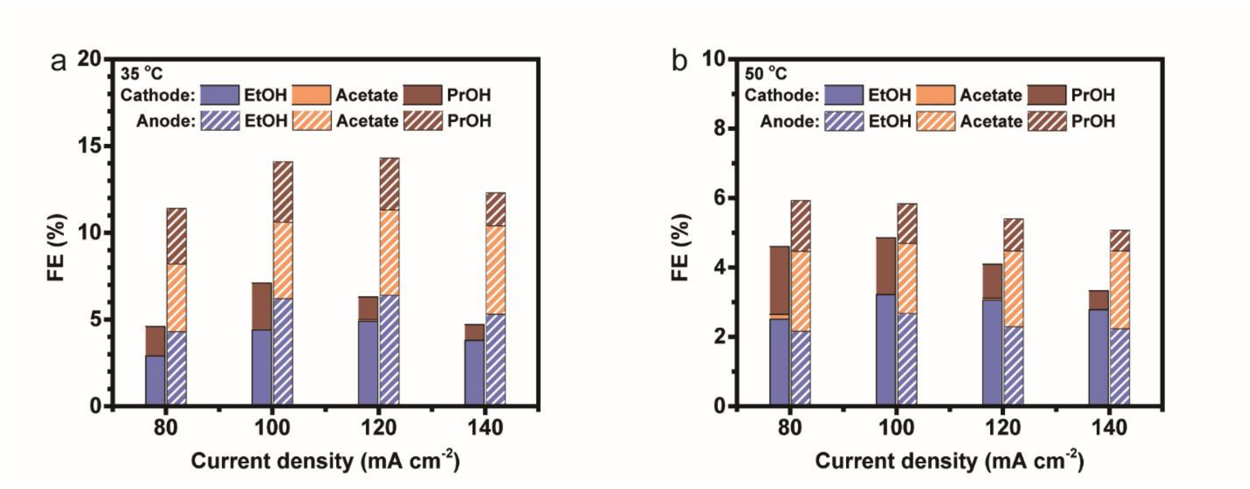

Fig. S8. The FE toward liquid product distributions in CO_2RR-GOR electrolyzer in the cathodic (solid) and anodic (patterned) streams at 35 ^{o}C (a) and 50 ^{o}C (b). At 50 ^{o}C, only < 6% FE of CO_2RR products crosses-over to the anolyte for all the current densities studied. The mass loadings on the cathode and anode are Cu: 0.5 mg cm^{-2} and Pt: 2 mg cm^{-2}.

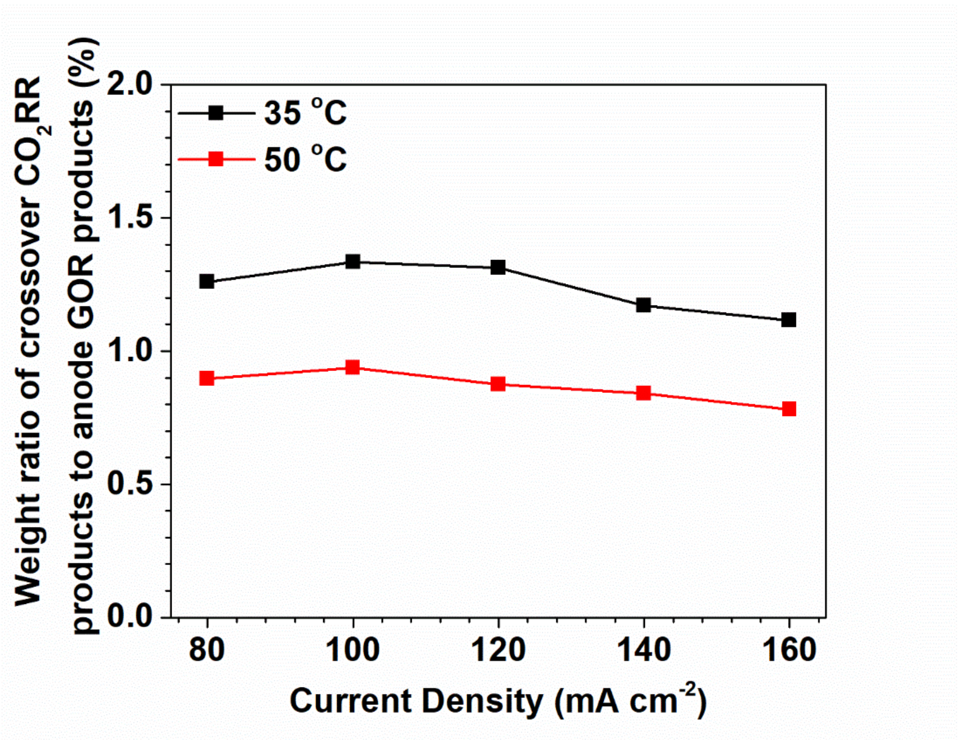

Fig. S9. The weight ratio between the liquid products of CO_2RR (ethanol, acetate and propanol) and the target products of GOR (gluconate, glucuronate and glucarate) at the temperature of 35 ^{o}C (black) and 50 ^{o}C (red).

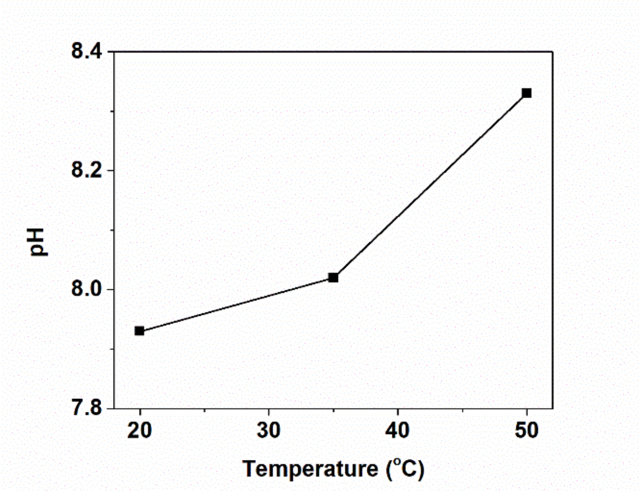

Fig. S10. The anolyte pH as a function of operating temperature. The anolyte type: 1 M $KHCO_3$ and 1 M glucose.

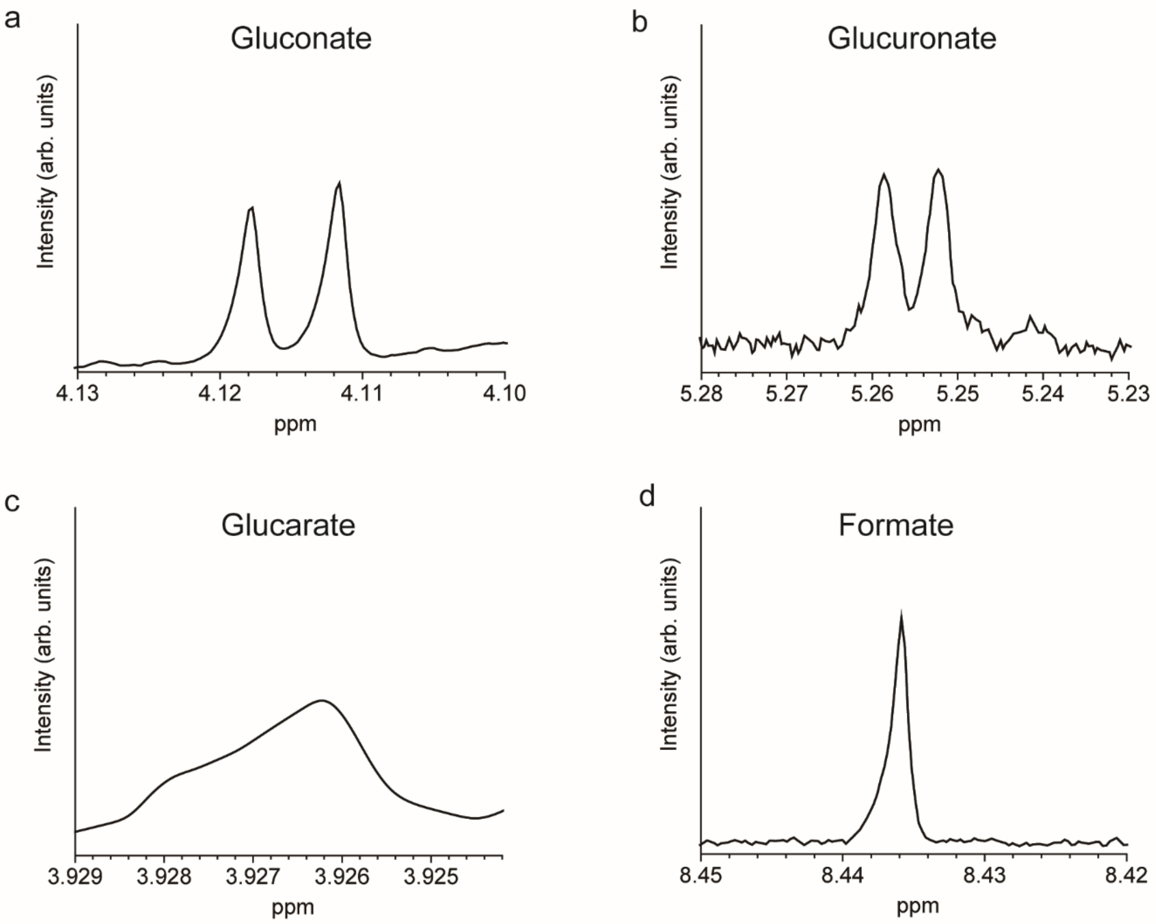

e

Species	Peak area	Proton number	Concentration (ppm)
DMSO (internal standard)	10,000	6	41.7
Gluconate	4,261	1	267.8
Glucuronate	523	1	32.5
Glucarate	852	2	9.2
Formate	491	1	7.2

Fig. S11. Representative of ^{1}H NMR spectra for quantifying the glucose oxidation reaction (GOR) products (a-d) in the anodic stream at 100 mA cm^{-2}. (e) The quantification of the GOR products based on the ^{1}H NMR spectra, with a 41.7 ppm DMSO as the internal standard. The concentration (ppm by mass) refers to the concentration in the NMR tube, which was 12x diluted by D_2O from the original solution.

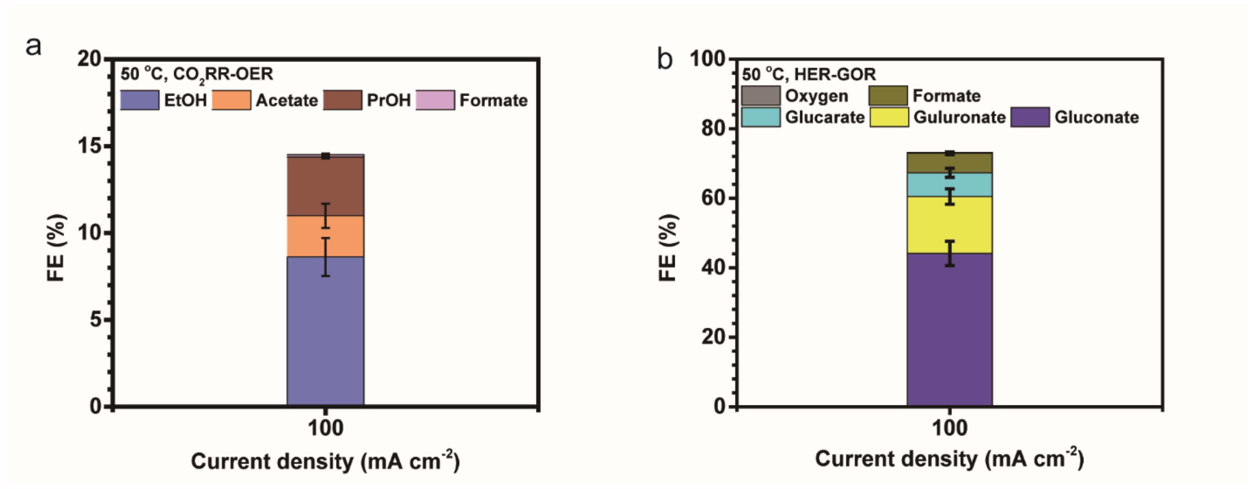

Fig. S12. The NMR-based analysis of liquid products from CO_2RR (a) and GOR (b). CO_2RR was coupled with OER, and GOR was coupled with HER to quantify their liquid-phase products separately.

CO_2RR can produce formate, migrate over the AEM and mix with that produced by GOR. The specific catalyst in this study has a formate FE of 0.12% (Fig. S12a), negligible to the 5.6% produced by GOR (Fig. S12b). We, therefore, assign the formate detected in the anolyte of the CO_2RR-GOR system to GOR. On the other hand, the other CO_2RR products – ethanol, propanol and acetate – are not detected in the GOR products.

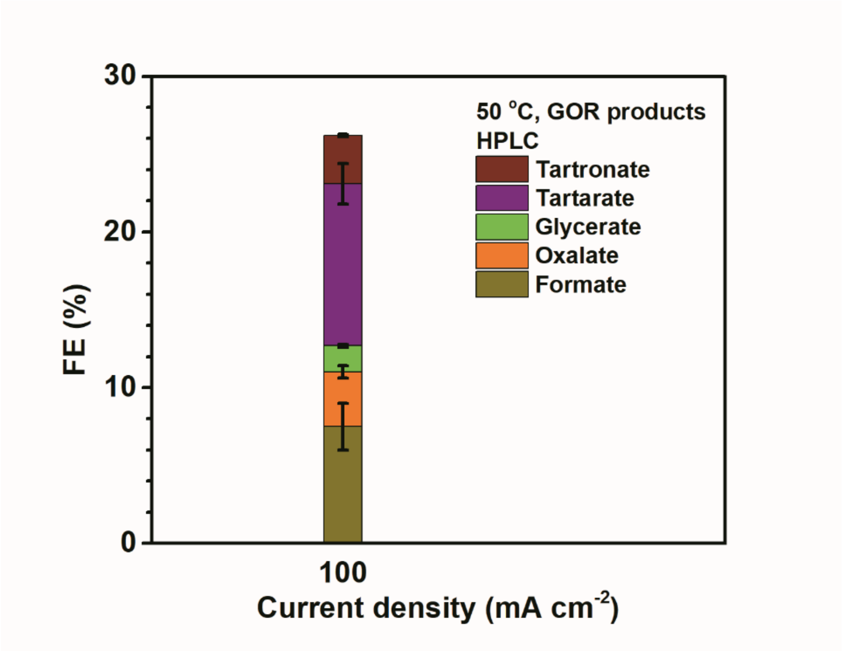

Fig. S13. The analysis of GOR by-products based on high-performance liquid chromatography (HPLC) analysis. The analyte was collected from an HER-GOR system (at 50°C, 100 mA cm^{-2}) to exclude any possible interference from CO_2RR liquid products. The peaks are identified referencing the handbook from the HPLC column producer (Aminex HPX-87H, BioRad), prior literature,[6–8] and the injection of standard samples. The main products – gluconate, glucaric and glucuronate peaks are covered by strong glucose signals thus cannot be quantified in HPLC.

In principle, other than gluconate, guluronate and glucarate, GOR can yield formate, oxalate, glycerate, tartarate and tartronate[6]. From ^{1}H NMR, we have quantified gluconate, guluronate, glucarate and formate while the other potential products were below our limit of detection. We performed HPLC to analyze the GOR products, and the results are shown in Fig. S13. The FE of formate agrees with the value calculated from ^{1}H NMR (Fig. S12b). The total FE summing over other by-products – oxalate, glycerate, tartarate and tartronate – is ~20% in total."

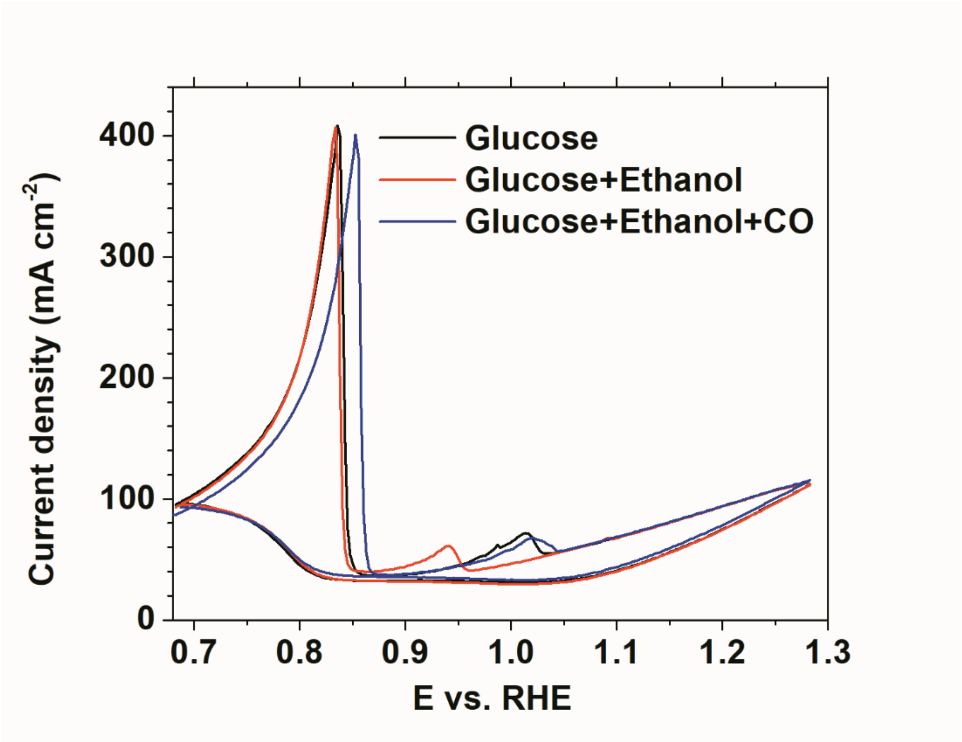

Fig. S14. The cyclic voltammetry (CV) profiles of the 2.0 mg cm^{-2} Pt/C on carbon paper anode in: i) 1 M $KHCO_3$ + 1 M glucose (black); ii) (i) + 200ppm ethanol (red); iii) (ii) saturated with CO. The profiles were recorded at a sweeping rate of 10 mV s^{-1}, in a single-component cell with Pt/C, graphite and AgCl/Ag as the working, counter and reference electrodes, respectively. We convert the voltage vs. AgCl/Ag to RHE using: $E_{RHE} = E_{Ag/AgCl} + 0.059\ pH + E^o_{Ag/AgCl}$, assuming $E^o_{Ag/AgCl}$ = 0.1976, and the pH was 8.2 as measured by a digital pH meter.

Pt catalyzed alcohol oxidation first to first to CO and then CO_2[22]. Ethanol is one of the main liquid products of the CO_2RR in this study. In typical experiments, the ethanol accumulated in the anolyte is in the range of 50 to 150 ppm. We performed CV studies on the Pt/C catalyst (Fig. S14) to investigate the CO2RR liquid products' impact on GOR. Adding 200 ppm ethanol into the electrolyte containing 1 M glucose, the CV profile alikes to that before adding ethanol (Fig. S14). Saturating the electrolyte with CO resulted in a similar CV profile. Considering that the ethanol oxidation peak typically appears at ~0.8 V vs. RHE, the present CVs indicate that the ethanol oxidation in the anodic stream of CO2RR-OER is insignificant. GOR predominates the anodic reaction owing to the high availability of glucose.

Table S4. The carbon isotope analysis of the anodic CO_2 flow.

Carbon source	***$\delta\ ^{13}C_{VPDB}$ (per mil)***
$KHCO_3$	-49.35 ± 0.12
CO_2 in cylinder	-43.13 ± 0.02
Glucose	-14.21 ± 2.06
Anodic gas stream	-49.87 ± 0.06

The abundance of ^{13}C is defined as $\delta\ ^{13}C_{VPDB}$[23]:

$$\delta^{13}C_{VPDB} = \left(\frac{\left(\frac{^{13}C}{^{12}C}\right)_{sample}}{\left(\frac{^{13}C}{^{12}C}\right)_{standard}} - 1\right) \times 1000‰$$

Where the standard ^{13}C:^{12}C ratio (Vienna Pee Dee Belemnite, PDB) is 0.0112372[23].

The carbon originated from different sources has different ^{13}C abundances. For example, the commercial CO_2 has a $\delta\ ^{13}C_{VPDB}$ value of -54‰ to -29‰, while the organisms have a $\delta\ ^{13}C_{VPDB}$ signature of about -25‰[23,24]. We, therefore, measured the carbon isotopes in bicarbonate, CO_2 in the cylinder, glucose and the CO_2 collected from the anodic gas stream to identify the source of the CO_2. As seen from the results listed in Table S4, the CO_2 collected from the anodic gas stream has a $\delta\ ^{13}C_{VPDB}$ very close to that detected in bicarbonate. We thus conclude that the majority of the anodic CO_2 originates from the acidification of bicarbonate, of which the carbon was supplemented by the crossover CO_2. The contribution of the glucose over oxidation to the anodic CO_2 – if any – is below 0.2% (the measurement error for $^{13}C/^{12}C$ of the anodic CO_2 gas sample is $\sim 7\times10^{-7}$). In light of the fact that: i) the sum of GOR FE from NMR and HPLC is close to 100%; ii) the Pt/C catalyst we are using is inactive to the alcohols under the operating conditions; iii) >99.8% of the anodic CO_2 gas stream comes from bicarbonate acidification, we conclude that the carbon efficiency and mass balance calculation in this work is valid and reliable.

Supplementary note 2:

The cathodic and anodic liquid-phase products are evaluated from the ^{1}H NMR spectra of catholyte and anolyte, respectively. The typical ^{1}H NMR spectra are shown in Fig. S11. Notably, formate can be detected in the anolyte which is majorly ascribed to the oxidation of glucose [25,26]. However, some of the formate may also come from the CO_2RR. In our previous studies [15], the Cu nanoparticle usually shows a low formate FE of < 1.5%.

Table S5. The CO_2RR gas product distribution of the copper-based catalyst in the MEA at various current densities at 20 ^oC. The mass loadings on the cathode and anode are Cu: 0.5 mg cm^{-2} and Pt: 2 mg cm^{-2}.

**Current density (mA cm^{-2})**	**Cell voltage (V)**	**Faradaic efficiency (%)**				**$J_{ethylene}$ (mA cm^{-2})**
		C_2H_4	CO	CH_4	H_2	
80	2.12±0.04	26.6	9.9	<0.1	29.3	21.3
100	2.41±0.02	41.8	6.2	<0.1	28.3	41.8
120	2.65±0.05	41.6	4.8	<0.1	26.2	49.9
140	2.85±0.08	41.6	1.9	<0.1	37.9	58.2

MEA operating conditions: anolyte: 1 M $KHCO_3$+1 M glucose; anolyte flow rate: 20 mL/min; CO_2 inlet flow rate: 10 sccm cm^{-2}; cell temperature: 20 ^oC; and cell pressure: atmospheric pressure.

Table S6. The CO_2RR product distribution of the copper-based catalyst in the MEA at various current densities at 35 ºC. The mass loadings on the cathode and anode are Cu: 0.5 mg cm^{-2} and Pt: 2 mg cm^{-2}.

Current density (mA cm^{-2})	Cell voltage (V)	Faradaic efficiency (%)*							$J_{ethylene}$ (mA cm^{-2})	J_{CO2RR} (mA cm^{-2})
		C_2H_4	EtOH	n-PrOH	Acetate	CO	CH_4	H_2		
80	2.00±0.05	42.1	7.2	4.9	3.9	2.9	<0.1	24.1	33.7	48.8
100	2.21±0.06	48.0	10.6	6.2	4.4	1.9	<0.1	33.0	48.0	71.1
120	2.30±0.04	41.8	11.3	4.3	5.0	1.4	<0.1	43.4	50.2	76.6
140	2.43±0.06	33.3	9.1	2.8	5.1	1.4	<0.1	46.4	46.7	72.5
160	2.55±0.03	26.9	8.9	2.6	4.1	1.1	<0.1	60.6	43.0	69.7

MEA operating conditions: anolyte: 1 M $KHCO_3$+1 M glucose; anolyte flow rate: 20 mL/min; CO_2 inlet flow rate: 10 sccm cm^{-2}; cell temperature: 35 ºC; and cell pressure: atmospheric pressure.

*The liquid product FEs account the detected in both cathodic and anodic streams.

Table S7. The glucose oxidation reaction (GOR) product distribution of the Pt-C catalyst in the MEA at various current densities and 35 ºC. The mass loadings on the cathode and anode are Cu: 0.5 mg cm^{-2} and Pt: 2 mg cm^{-2}.

Current density (mA cm^{-2})	Cell voltage (V)	Faradaic efficiency (%)					$J_{gluconate}$ (mA cm^{-2})
		Gluconate	Formate	Glucarate	Glucuronate	Total*	
80	2.00±0.05	34.2	6.8	6.1	18.7	65.8	27.4
100	2.21±0.06	43.6	3.9	5.4	13.6	66.5	43.6
120	2.30±0.04	48.1	3.2	4.8	11.2	67.3	57.7
140	2.43±0.06	51.4	2.5	3.9	7.4	65.2	72.0
160	2.55±0.03	46.7	2.4	2.7	4.3	56.1	74.7

MEA operating conditions: anolyte: 1 M $KHCO_3$ + 1 M glucose; anolyte flow rate: 20 mL/min; cell temperature: 35 ºC; and cell pressure: atmospheric pressure.

*The quantification is based on NMR. Some of the GOR products, such as oxalate, tartronicate, tartarate and glycorate, can hardly be quantified in NMR. According to the HPLC evaluation (Fig. S13), accounting all these products, the total anodic FE is close to 100%.

Table S8. The CO_2RR product distribution of the copper-based catalyst in the MEA at various current densities at 50 °C. The mass loadings on the cathode and anode are Cu: 0.5 mg cm^{-2} and Pt: 2 mg cm^{-2}.

Current density (mA cm^{-2})	Cell voltage (V)	Faradaic efficiency (%)*							$J_{ethylene}$ (mA cm^{-2})	J_{CO2RR} (mA cm^{-2})
		C_2H_4	EtOH	n-PrOH	Acetate	CO	CH_4	H_2		
80	1.76±0.02	33.9	4.8	3.2	2.8	8.3	<0.1	33.9	27.1	43.2
100	1.81±0.04	43.5	6.0	2.2	2.2	12.0	<0.1	43.5	43.5	64.5
120	2.08±0.02	41.0	5.5	1.3	2.0	11.1	<0.1	41.0	49.2	72.2
140	2.26±0.04	38.6	5.2	1.5	2.2	6.6	<0.1	38.6	54.1	72.4
160	2.40±0.03	34.1	4.1	1.1	2.0	3.9	<0.1	34.1	54.6	72.4

MEA operating conditions: anolyte: 1 M $KHCO_3$+1 M glucose; anolyte flow rate: 20 mL/min; CO_2 inlet flow rate: 10 sccm cm^{-2}; cell temperature: 50 °C; and cell pressure: atmospheric pressure.
*The liquid product FEs account the detected in both cathodic and anodic streams.

Table S9. The glucose oxidation reaction (GOR) product distribution of the Pt-C catalyst in the MEA at 50 ^{o}C and various current densities. The mass loadings on the cathode and anode are Cu: 0.5 mg cm^{-2} and Pt: 2 mg cm^{-2}.

Current density (mA cm^{-2})	Cell voltage (V)	Faradaic efficiency (%)					$J_{gluconate}$ (mA cm^{-2})
		Gluconate	Formate	Glucarate	Glucuronate	Total*	
80	1.76±0.02	38.3	8.3	5.6	25.3	71.3	37.1
100	1.81±0.04	49.4	4.4	5.8	15.9	74.1	48.6
120	2.08±0.02	49.1	5.2	6.1	13.8	73.9	62.8
140	2.26±0.04	57.7	4.9	3.6	8.2	69.6	78.8
160	2.40±0.03	58.5	2.5	2.2	3.2	64.8	89.9

MEA operating conditions: anolyte: 1 M $KHCO_3$+1 M glucose; anolyte flow rate: 20 mL/min; cell temperature: 50 ^{o}C; and cell pressure: atmospheric pressure.

*The quantification is based on NMR. Some of the GOR products, such as oxalate, tartronicate, tartarate and glycorate, can hardly be quantified in NMR. According to the HPLC evaluation (Fig. S13), accounting all these products, the total anodic FE is close to 100%.

Table S10. The CO_2RR product distribution of the copper-based catalyst in the MEA at 100 mA cm^{-2} at various CO_2 flow rates. The mass loadings on the cathode and anode are Cu: 0.5 mg cm^{-2} and Pt: 2 mg cm^{-2}.

**CO_2 flow rate (sccm cm^{-2})**	**Cell voltage (V)**	**Faradaic efficiency (%)***							**Carbon efficiency**	**CO_2-to-C_2H_4 conversion**
		**C_2H_4**	**EtOH**	**n-PrOH**	**Acetate**	**CO**	**CH_4**	**H_2**		
0.18	1.9±0.1	25.7	5.8	1.1	1.3	5.1	4.8	46.1	75.3	36
0.36	1.9±0.1	32.2	8.4	1.1	3.5	7.1	3.4	42.2	48.5	23
0.96	1.9±0.1	36.3	10.4	1.9	1.6	8.1	2.3	37.6	20.3	9.5
1.46	1.9±0.1	39.4	10.9	1.8	1.6	9.3	1.5	33.2	14.4	6.8

MEA operating conditions: anolyte: 1 M $KHCO_3$+1 M glucose; anolyte flow rate: 20 mL/min; cell temperature: 50 ^{o}C; and cell pressure: atmospheric pressure.
*The liquid product FEs account the detected in both cathodic and anodic streams.

Table S11. The glucose oxidation reaction (GOR) product distribution of the Pt-C catalyst in the MEA at 100 mA cm^{-2} at various CO_2 flow rates. The mass loadings on the cathode and anode are Cu: 0.5 mg cm^{-2} and Pt: 2 mg cm^{-2}.

CO_2 flow rate (sccm cm^{-2})	Cell voltage (V)	Faradaic efficiency (%)					$J_{gluconate}$ (mA cm^{-2})
		Gluconate	Formate	Glucarate	Glucuronate	Total*	
0.18	1.9±0.1	57.6	6.6	6.5	15.6	86.3	57.6
0.36	1.9±0.1	52.9	7.1	6.3	13.1	80.8	52.9
0.96	1.9±0.1	55.0	5.9	5.8	12.7	79.4	55.0
1.46	1.9±0.1	56.9	6.0	5.9	14.4	83.2	56.9

MEA operating conditions: anolyte: 1 M $KHCO_3$+1 M glucose; anolyte flow rate: 20 mL/min; cell temperature: 50 ^{o}C; and cell pressure: atmospheric pressure.

*The quantification is based on NMR. Some of the GOR products, such as oxalate, tartronicate, tartarate and glycorate, can hardly be quantified in NMR. According to the HPLC evaluation (Fig. S13), accounting all these products, the total anodic FE is close to 100%.

Table S12. The CO_2RR product distribution of the copper-based catalyst in the MEA during extended operation. Current density: 100 mA/cm^2. Temperature: 50 ^{o}C. The mass loadings on the cathode and anode are Cu: 0.5 mg cm^{-2} and Pt: 2 mg cm^{-2}.

**Time (hour)**	**Faradaic efficiency (%)***							**$J_{ethylene}$ (mA cm^{-2})**	**J_{CO2RR} (mA cm^{-2})**	***Carbon efficiency (%)***
	**C_2H_4**	**EtOH**	**n-PrOH**	**Acetate**	**CO**	**CH_4**	**H_2**			
15	32.2	8.4	1.1	3.5	7.1	3.4	42.2	32.2	55.7	48.0
28	32.7	9.1	1.2	1.9	6.7	3.6	42.9	32.7	55.2	46.4
42	31.3	9.4	1.1	2.5	6.1	3.8	43.6	31.3	54.2	44.9
56	30.8	8.6	1.0	1.8	5.8	4.1	44.1	30.8	52.1	42.6
71	30.1	9.1	0.9	1.6	5.6	4.4	44.5	30.1	51.7	41.7

MEA operating conditions: anolyte: 1 M $KHCO_3$+1 M glucose; anolyte flow rate: 20 mL/min; cell temperature: 50 ^{o}C; CO_2 inlet flow rate: 0.36 sccm cm^{-2}; and cell pressure: atmospheric pressure.
*The liquid product FEs account the detected in both cathodic and anodic streams.

Table S13. The glucose oxidation reaction (GOR) product distribution of the Pt-C catalyst in the MEA during extended operation. Current density: 100 mA/cm^2. Temperature: 50 ºC. The mass loadings on the cathode and anode are Cu: 0.5 mg cm^{-2} and Pt: 2 mg cm^{-2}.

Time (hour)	Faradaic efficiency (%)					$J_{gluconate}$ (mA cm^{-2})
	Gluconate	Formate	Glucarate	Glucuronate	Total*	
15	49.7	7.6	8.1	14.4	79.8	49.7
28	47.3	8.2	8.9	12.8	77.2	47.3
42	45.0	8.1	9.5	13.5	76.1	45.0
56	52.5	8.4	10.3	13.4	84.6	52.5
71	45.0	7.2	11.6	13.8	77.6	45.0

MEA operating conditions: anolyte: 1 M $KHCO_3$+1 M glucose; anolyte flow rate: 20 mL/min; CO_2 inlet flow rate: 0.36 sccm cm^{-2}; cell temperature: 50 ºC; and cell pressure: atmospheric pressure.

*The quantification is based on NMR. Some of the GOR products, such as oxalate, tartronicate, tartarate and glycorate, can hardly be quantified in NMR. According to the HPLC evaluation (Fig. S13), accounting all these products, the total anodic FE is close to 100%.

Table S14 The comparison of the energy intensities between CO_2RR-OER and CO_2RR-GOR systems operating at different current densities, based on the experimental results shown in Fig. 3d (CO_2RR-GOR) and Fig. S3b (CO_2RR-OER, the crossover CO_2 was not recirculated). This comparison assumes the CO_2 in anodic gas stream in CO_2RR-OER system is isolated from O_2 then redirected to the gas feeding since 33% O_2 reduces the CO_2RR selectivity to ~0%. The anodic gas stream of the CO_2RR-GOR system is co-fed with the input CO_2 stream.

Configuration	Current denisty (mA cm^{-2})	80	100	120	140	160
CO_2RR-OER (50 ^oC)	Cell voltage (V)	3.07	3.18	3.24	3.33	3.44
	Ethylene FE (%)	34.8	46.5	42.0	31.6	31.1
	O_2% in anodic gas stream	33	33	33	33	33
	Electricity (GJ/ton ethylene)	365	283	319	436	457
	Anode gas separation (GJ/ton ethylene)	92	69	76	30	103
	**Energy intensity (excl. cathode separation, GJ/ton ethylene)**	**457**	**352**	**395**	**466**	**560**
CO_2RR-GOR (50 ^oC)	Cell voltage (V)	1.76	1.81	2.08	2.26	2.40
	Ethylene FE (%)	33.8	43.5	41.0	38.6	34.1
	O_2% in anodic gas stream	0.40	0.40	0.01	1.4	1.2
	Electricity (GJ/ton ethylene)	215	172	210	242	291
	Anode gas separation (GJ/ton ethylene)	0	0	0	0	0
	**Energy intensity (excl. cathode separation, GJ/ton ethylene)**	**215**	**172**	**210**	**242**	**291**
The cathodic gas separation energy is not included because at a specific current density, the CO_2RR-OER and CO_2RR-GOR systems show similar ethylene FEs. We assume that their ethylene FE vs. carbon efficiency diagram have a similar trend, so as their cathodic separation requirements.						

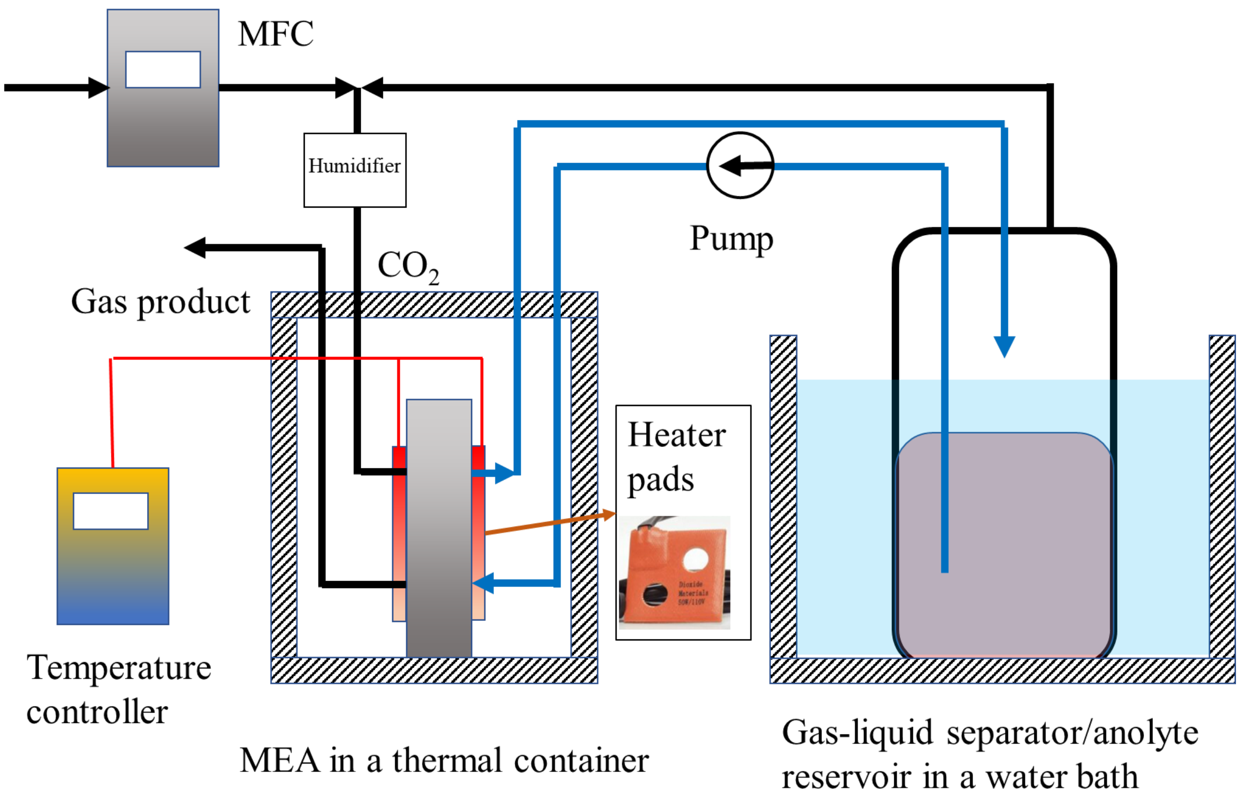

Fig. S15. The schematic of the auxiliary system for heating the CO_2RR-OOR electrolyzer. In this system, all the pipes and gas lines were covered by homemade thermal insulation layers composed of cotton-lined aluminum foil. The flow rate of the input CO_2 was controlled by a mass flow controller (MFC). The MEA was heated by a pair of heater pads purchased from Dioxide Materials, and the pads were connected to a homemade temperature controller. Water baths controlled the temperature of the anolyte reservoir and the humidifier.

References

1. Gabardo, C. M. *et al.* Continuous Carbon Dioxide Electroreduction to Concentrated Multi-carbon Products Using a Membrane Electrode Assembly. *Joule* **3**, 2777–2791 (2019).

2. Li, F. *et al.* Molecular tuning of CO2-to-ethylene conversion. *Nature* **577**, 509–513 (2020).

3. House, K. Z. *et al.* Economic and energetic analysis of capturing CO2 from ambient air. *Proc. Natl. Acad. Sci. U. S. A.* **108**, 20428–20433 (2011).

4. Song, C., Liu, Q., Deng, S., Li, H. & Kitamura, Y. Cryogenic-based CO2 capture technologies: State-of-the-art developments and current challenges. *Renew. Sustain. Energy Rev.* **101**, 265–278 (2019).

5. Gao, H. *et al.* Comparative studies of heat duty and total equivalent work of a new heat pump distillation with split flow process, conventional split flow process, and conventional baseline process for CO2 capture using monoethanolamine. *Int. J. Greenh. Gas Control* **24**, 87–97 (2014).

6. Plaza, J. M., Wagener, D. Van & Rochelle, G. T. Modeling CO2 capture with aqueous monoethanolamine. *Energy Procedia* **1**, 1171–1178 (2009).

7. Tuinier, M. J., Van Sint Annaland, M. & Kuipers, J. A. M. A novel process for cryogenic CO 2 capture using dynamically operated packed beds-An experimental and numerical study. *Int. J. Greenh. Gas Control* **5**, 694–701 (2011).

8. Tuinier, M. J., Hamers, H. P. & Van Sint Annaland, M. Techno-economic evaluation of cryogenic CO2 capture-A comparison with absorption and membrane technology. *Int. J. Greenh. Gas Control* **5**, 1559–1565 (2011).

9. Song, C. F., Kitamura, Y., Li, S. H. & Jiang, W. Z. Parametric Analysis of a Novel Cryogenic CO2 Capture System Based on Stirling Coolers. *Environ. Sci. Technol.* **46**, 12735–12741 (2012).

10. Keith, D. W., Holmes, G., St. Angelo, D. & Heidel, K. A Process for Capturing CO2 from the Atmosphere. *Joule* **2**, 1573–1594 (2018).

11. Carolina Font-Palma, David Cann, C. U. Review of Cryogenic Carbon Capture Innovations and Their Potential Applications. *J. Carbon Res.* **7**, 58 (2021).

12. Rao, A. B. & Rubin, E. S. Identifying cost-effective CO 2 control levels for amine-based CO 2 capture systems. *Ind. Eng. Chem. Res.* **45**, 2421–2429 (2006).

13. Sisler, J., Shaihroz Kha, Ip, A. H., Jaffer, M. W. S. S. A. & Sargent, E. H. Ethylene Electrosynthesis : A Comparative Techno-economic Analysis of Alkaline vs Membrane Electrode Assembly vs CO2−CO− C2H4 Tandems. *ACS Energy Lett.* **6**, 997–1002 (2021).

14. Sheng, W., Myint, M., Chen, J. G. & Yan, Y. Correlating the hydrogen evolution reaction activity in alkaline electrolytes with the hydrogen binding energy on monometallic

surfaces. *Energy Environ. Sci.* **6**, 1509–1512 (2013).

15. Ozden, A. *et al.* High-rate and efficient ethylene electrosynthesis using a catalyst/promoter/transport layer. *ACS Energy Lett.* **5**, 2811–2818 (2020).

16. Huang, J. E. *et al.* CO 2 electrolysis to multi-carbon products in strong acid. *Science* **372**, 1074–1078 (2021).

17. Prices of chemical elements. *https://en.wikipedia.org/wiki/Prices_of_chemical_elements*

18. Liu, W. J. *et al.* Efficient electrochemical production of glucaric acid and H2 via glucose electrolysis. *Nat. Commun.* **11**, 1–11 (2020).

19. Moggia, G., Kenis, T., Daems, N. & Breugelmans, T. Electrochemical Oxidation of d-Glucose in Alkaline Medium: Impact of Oxidation Potential and Chemical Side Reactions on the Selectivity to d-Gluconic and d-Glucaric Acid. *ChemElectroChem* **7**, 86–95 (2020).

20. Solmi, S., Morreale, C., Ospitali, F., Agnoli, S. & Cavani, F. Oxidation of d-Glucose to Glucaric Acid Using Au/C Catalysts. *ChemCatChem* **9**, 2797–2806 (2017).

21. A. Larew, L. & Johnson, D. C. Concentration dependence of the mechanism of glucose oxidation at gold electrodes in alkaline media. *J. Electroanal. Chem.* **262**, 167–182 (1989).

22. Marinkovic, N. S., Li, M. & Adzic, R. R. *Pt-Based Catalysts for Electrochemical Oxidation of Ethanol*. *Topics in Current Chemistry* **377**, (Springer International Publishing, 2019).

23. δ13C. *https://en.wikipedia.org/wiki/Δ13C* Available at: https://en.wikipedia.org/wiki/Δ13C.

24. Natural Variations of Isotopic Abundances. *https://www.ciaaw.org/natural-variations.htm?el=carbon*

25. Tominaga, M., Shimazoe, T., Nagashima, M. & Taniguchi, I. Composition-activity relationships of carbon electrode-supported bimetallic gold-silver nanoparticles in electrocatalytic oxidation of glucose. *J. Electroanal. Chem.* **615**, 51–61 (2008).

26. Tominaga, M., Shimazoe, T., Nagashima, M. & Taniguchi, I. Electrocatalytic oxidation of glucose at gold nanoparticle-modified carbon electrodes in alkaline and neutral solutions. *Electrochem. commun.* **7**, 189–193 (2005).
